# Structural determinants for red‐shifted absorption in higher‐plants Photosystem I

**DOI:** 10.1111/nph.70562

**Published:** 2025-09-15

**Authors:** Stefano Capaldi, Zeno Guardini, Daniele Montepietra, Vittorio Flavio Pagliuca, Antonello Amelii, Elena Betti, Chris John, Laura Pedraza‐González, Lorenzo Cupellini, Benedetta Mennucci, Diane Marie Valerie Bonnet, Antonio Chaves‐Sanjuan, Luca Dall'Osto, Roberto Bassi

**Affiliations:** ^1^ Dipartimento di Biotecnologie Università di Verona Strada Le Grazie 15 37134 Verona Italy; ^2^ Dipartimento di Chimica e Chimica Industriale Università di Pisa Via Giuseppe Moruzzi 13 56124 Pisa Italy; ^3^ Dipartimento di Bioscienze Università di Milano Via Celoria 26 20133 Milan Italy; ^4^ Fondazione Romeo e Enrica Invernizzi and Unitech NOLIMITS Università di Milano Via Celoria 26 20133 Milan Italy; ^5^ Accademia Nazionale dei Lincei, Palazzo Corsini Via della Lungara 10 00165 Rome Italy; ^6^ Anton Dorhn Experimental Marine Station Villa Comunale 80121 Naples Italy

**Keywords:** far‐red, Lhca, light‐harvesting, low‐energy absorption, photosynthesis, Photosystem I, red forms

## Abstract

- Higher plants Photosystem I absorbs far‐red light, enriched under vegetation canopies, through long‐wavelength Chls to enhance photon capture. Far‐red absorption originates from Chl pairs within the Lhca3 and Lhca4 subunits of the LHCI antenna, known as the ‘red cluster’, including Chls *a*603 and *a*609.
- We used reverse genetics to produce an *Arabidopsis* mutant devoid of red‐shifted absorption, and we obtained high‐resolution cryogenic electron microscopy structures of PSI‐LHCI complexes from both wild‐type and mutant plants.
- Computed excitonic coupling values suggested contributions from additional nearby pigment molecules, namely Chl *a*615 and violaxanthin in the L2 site, to far‐red absorption. We investigated the structural determinants of far‐red absorption by producing further *Arabidopsis* transgenic lines and analyzed the spectroscopic effects of mutations targeting these chromophores. The two structures solved were used for quantum mechanics calculations, revealing that excitonic interactions alone cannot explain far‐red absorption, while charge transfer states were needed for accurate spectral simulations.
- Our findings demonstrate that the molecular mechanisms of light‐harvesting under shaded conditions rely on very precise tuning of chromophore interactions, whose understanding is crucial for designing light‐harvesting complexes with engineered absorption spectra.

## Introduction

Photosynthetic organisms use solar radiation as their primary energy source to convert carbon dioxide and water into oxygen and sugars. The light reactions of oxygenic photosynthesis involve two multimeric pigment‐binding protein complexes: photosystems (PS) I and II, which work in series. The initial step of light harvesting is catalyzed by PSII, which is responsible for water splitting and oxygen evolution. Meanwhile, PSI mediates the reduction of NADP^+^ to NADPH, serving as a temporary storage for reducing power (Croce & van Amerongen, 2020). PSs share a common structure that includes a core complex housing the reaction centers (RC) where charge separation reactions occur and an antenna system (Light Harvesting Complexes (LHC) enhancing the light‐absorbing cross‐section of the complexes (Pan *et al*., 2020)). Despite these similarities, there are striking differences in their spectral properties, with PSI exhibiting a red‐shifted absorption profile (Rivadossi *et al*., 1999). The red‐most PSI absorption arises from special Chl pairs that absorb at lower energies than RC P700, referred to as ‘red Chl forms’ or simply ‘red forms’ (RF; Croce & Van Amerongen, 2013).

These pigments extend the absorption capacity into the far‐red spectrum (λ ≥ 700 nm), providing advantages under canopy or in dense culture conditions where most visible wavelengths are absorbed by the upper leaf layers (Martínez‐García *et al*., 2010) while far‐red radiation is strongly enriched, resulting in a far‐red to red ratio higher than 5 (Park & Runkle, 2017). It is worth noting that, although RF contribute only a small percentage of the total absorption (Gobets & Van Grondelle, 2001), they are preferentially excited under far‐red enriched light conditions (Croce *et al*., 1998; Rivadossi *et al*., 1999). Moreover, RF are highly effective in energy transfer and trapping, as *c*. 80% of the PSI excitation transits through these pigments to reach P700 (Croce *et al*., 1998). Since the excited states associated with these low‐energy Chls are highly populated, they may play crucial roles in photoprotection and/or the concentration of excitation energy (Rivadossi *et al*., 1999; Jennings *et al*., 2003; Carbonera *et al*., 2005). However, their exact physiological role(s) remain to be fully understood (Jennings *et al*., 2013).

**Fig. 1:**
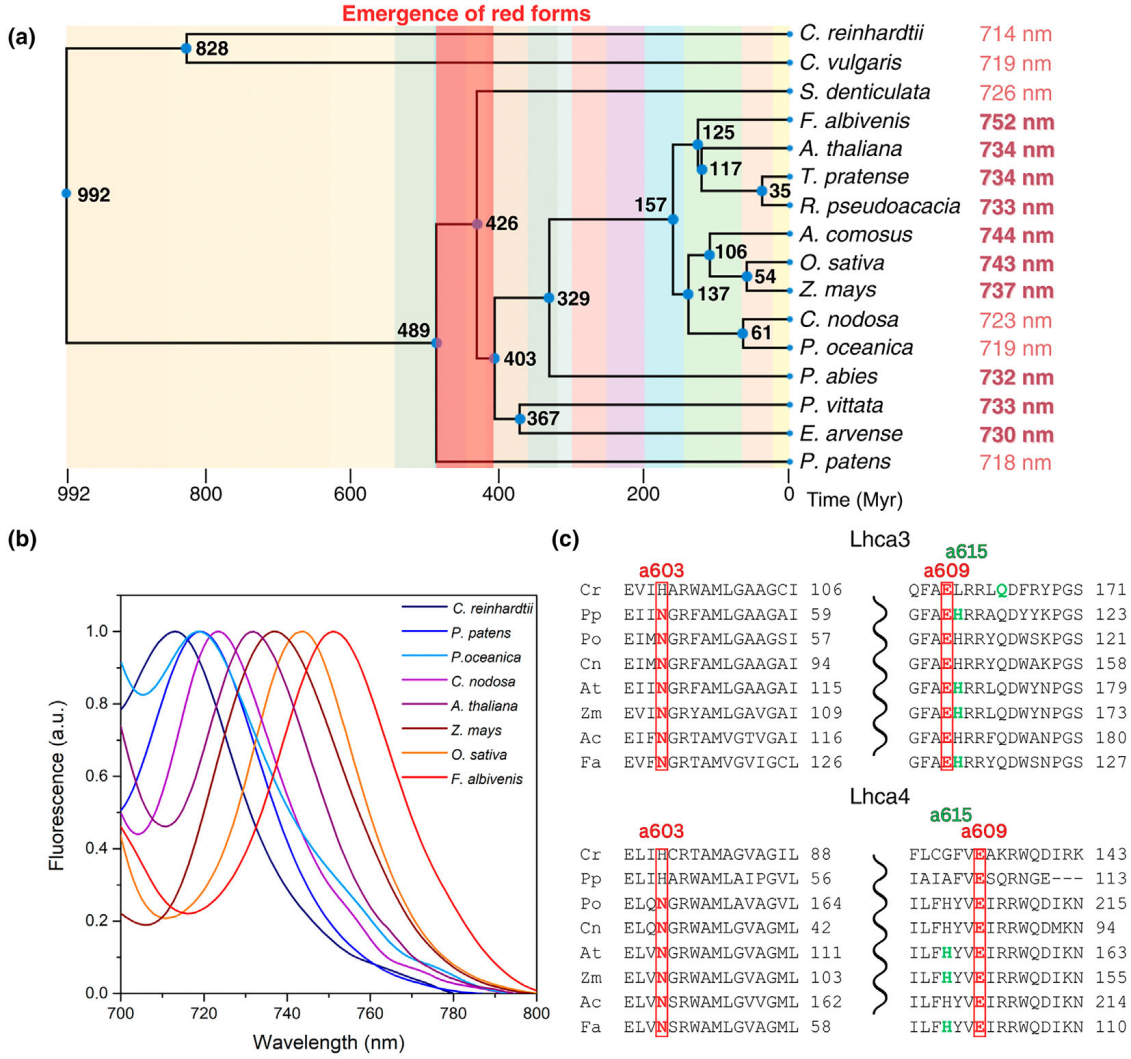
Evolution of low‐absorbing spectral features in Viridiplantae. (a) Time tree of representative Viridiplantae species and their divergence times (in million years, Myr). The most red‐shifted fluorescence emission peak of each species' experimental spectra is highlighted in bold. Note that the PSI core complex emits at *c*. 720 nm. Thus, red forms associated with LHCI proteins can be readily detected only when emitting at >720 nm. The emergence of red forms (RF) can be traced back to *c*. 489–403 Myr co‐eval with the appearance of tall trees and canopies. (b) Low temperature (77 K) fluorescence emission spectra of representative species from green algae (*Chlamydomonas reinhardtii*), mosses (*Physcomitrium patens*), and full sun land plant angiosperms: *Zea mays*, *Oryza sativa* (*Poaceae*) and *Arabidopsis thaliana* (*Brassicaceae*); shade plants: *Fittonia albivenis* (*Acanthaceae*); and seagrasses (*Posidonia oceanica* and *Cymodocea nodosa*). Shade plants thrive in far‐red enriched light, while seagrasses thrive in the absence of far‐red radiation. (c) Multiple alignments of the Lhca4 (corresponding to Lhca8 for *C. reinhardtii* and Lhca2b for *P. patens* and Lhca3 protein sequences from *C. reinhardtii* (Cr), *P. patens* (Pp)), *P. oceanica* (Po), *C. nodosa* (Cn), *A. thaliana* (At), *Z. mays* (Zm), *Ananas comosus* (Ac), and *F. albivenis* (Fa). Residues binding red form Chls (Chl *a*603 and Chl *a*609) are colored in red and shown in red rectangles. Coordinating residues for Chl *a*615 are colored green for species with reported PDB structures. Details about the acquisition of the time tree in panel (a) and the spectra in panel (b) are reported in the Supporting Information. PS, photosystem; LHC, light harvesting complex.

RF are present in nearly all types of PSI complexes, and their occurrence is closely related to the availability of far‐red radiation. In cyanobacteria (e.g. *Arthrospira platensis*), these pigments are localized within the core complex and allow for the absorption of far‐red photons, resulting in PSI low temperature fluorescence emission peaks between 730 and 760 nm (Rakhimberdieva *et al*., 2001). In green algae (e.g. *Chlamydomonas reinhardtii*) and mosses (e.g. *Physcomitrium patens*), red Chls are associated with the LHCI antenna system, resulting in a PSI‐LHCI fluorescence emission peak wavelength *c*. 715 and 722 nm, respectively (Mozzo *et al*., 2010; Gorski *et al*., 2022; Fig. 1). Upon land colonization, environmental niches enriched in far‐red radiation became widespread under canopy, resulting in a progressively increased red‐edge Q_Y_ absorption associated with LHCI (Croce *et al*., 1998). Indeed, the PSI‐LHCI fluorescence emission from flowering plants leaves shifts toward the red, peaking at 730 nm or even beyond (Akhtar & Lambrev, 2020; Bos *et al*., 2023; Li *et al*., 2024).

In higher plants, LHCI binds the PSI core complex as functional heterodimers: Lhca1‐Lhca4 and Lhca2‐Lhca3, both of which exhibit similar spectral properties and emit far‐red fluorescence at *c*. 730 nm (Wientjes & Croce, 2011). *In vitro* reconstitution of recombinant Lhca complexes (rLhca) showed that the far‐red shift is primarily caused by the Lhca4 and Lhca3 components of the dimers (Croce *et al*., 2002; Castelletti *et al*., 2003), and is linked to a specific sequence substitution unique to these two LHCs, where an Asn serves as the binding residue for Chl *a*603 instead of a His residue, as seen in all other LHC proteins (Jansson, 1999; Morosinotto *et al*., 2003; Fig. 1c). Indeed, the substitution of Asn (N) with His (H) (*a*603‐NH substitution) resulted in the complete loss of the far‐red absorption and emission forms in both rLhca3 and rLhca4 complexes (Morosinotto *et al*., 2003). It was hypothesized that the presence of Asn promotes strong excitonic interactions between the Chl *a*603–*a*609 pair (A5 and B5 according to Kühlbrandt's nomenclature; Kühlbrandt *et al*., 1994) leading to a charge transfer (CT) character (Romero *et al*., 2009) and ultimately resulting in the formation of the RF (Morosinotto *et al*., 2003). Complementing *koLhca4 Arabidopsis* lines with mutant Lhca4, carrying either His or nonpigment‐binding residues at the Chl *a*603 ligand position (Li *et al*., 2023) resulted in a blue shift of *c*. 3 nm at 77 K.

However, accumulating evidence suggests that the substitution of Asn vs His as the ligand of Chl *a*603 alone cannot account for the variability in far‐red spectral properties found in nature (Li *et al*., 2023; Elias *et al*., 2024). For instance, in the Lhca2/a4/a9 subunits of *C. reinhardtii*, where the Chl *a*603 ligand is Asn, emission peaks range between 690 and 717 nm, with significantly lower absorption beyond 700 nm compared with higher plants Lhca4 (Mozzo *et al*., 2010). Moreover, the *a*603‐HN (H111N) mutation, although introducing a significant red‐shift in the PSII antenna complex Lhcb4 (from 680 to 682 nm), did not achieve a shift as large as the 40 nm one observed in the Chl *a*603‐NH mutant of Lhca3 or Lhca4 (from *c*. 730 to *c*. 690 nm; Morosinotto *et al*., 2003; Guardini *et al*., 2020; Sardar *et al*., 2024), despite the high structural similarity between the antenna complexes.

In addition to the arrangement of relevant Chls, the surrounding microenvironment can be important for optimizing low‐energy spectral forms. Structural models of PSI‐LHCI reveal a chromophore cluster comprising three Chls – Chl *a*603, Chl *a*609, and Chl *a*615 – and two xanthophylls (Xan) – violaxanthin (Vio) in site L2 and a lutein (Lut; Qin *et al*., 2015). Notably, Chl *a*615 is present only in the Lhca3 and Lhca4 subunits – the red‐shifted subunits – while it is absent in all other LHC proteins, regardless of whether they serve PSI or PSII. This observation raised the question of its potential role in forming excitonic interactions (Melkozernov & Blankenship, 2003; Wientjes *et al*., 2012).

In this study, we investigated the structural and biophysical determinants of the red Chl forms in *Arabidopsis thaliana* PSI‐LHCI by combining *in vivo* site‐directed mutagenesis and single‐particle cryogenic electron microscopy (Cryo‐EM). We obtained two high‐resolution structures of the PSI‐LHCI supercomplexes from *A. thaliana* wild‐type (WT) and the *a*603‐NH mutant genotype, which lacks RF. We then computed the excitonic interactions within PSI‐LHCI pigments, focusing on the interaction between the L2 Xan and the Chl *a*603–*a*609 pair in forming low‐lying energy spectral states.

## Materials and Methods

### Plant materials

The *Arabidopsis thaliana* (L.) *Heynh koLhca3 koLhca4* mutant was generated by crossbreeding *koLhca3* and *koLhca4* NASC insertional lines, following the methodology outlined by Bressan *et al*. (2016). Complementation of the *koLhca3 koLhca4* mutant was achieved through *Agrobacterium tumefaciens*‐mediated transformation, as described by Zhang *et al*. (2006), resulting in *a*603‐NH, *a*615‐HA, *a*615‐HI, *A3WT‐A4WT*, *A3NH‐A4WT*, and *A3WT‐A4NH* lines.

### Purification and characterization of PSI‐LHCI samples

To purify PSI‐LHCI supercomplexes, *A. thaliana* plants were grown for *c*. 6 wk in a phytotron (150 μmol photons m^−2^ s^−1^, 23°C, 70% relative humidity, 8 h : 16 h, day : night). Before the isolation procedure, *Arabidopsis* leaves were dark‐adapted for 60 min at 4°C. Unstacked thylakoid membranes were prepared as described in Bassi *et al*. (1985). Thylakoid membranes were resuspended to a Chl concentration of 1 mg ml^‐1^ in 10 mM HEPES pH 7.5 and solubilized by adding an equal volume of 2% dodecyl‐β‐d‐maltoside (β‐DM). The samples were vortexed for 30 s and incubated on ice for 10 min, and the insoluble material was removed by centrifugation at 20 000 ***g*** for 10 min at 4°C. The supernatants were fractionated by sucrose gradient ultracentrifugation at 284 000 ***g*** at 4°C for 18 h (Beckman SW40 Ti rotor, Beckman Coulter, Brea, CA, USA) or at 141 000 ***g*** at 4°C for 30 h (Beckman SW28 Ti; Supporting Information Fig. S1). Bands containing PSI‐LHCI were harvested using a Hamilton syringe.

For Cryo‐EM preparations, PSI‐LHCI samples were concentrated to a final volume of *c*. 800 μl, and sucrose was removed by dialysis overnight against a solution containing 10 mM HEPES pH 7.5 and 0.05% β‐DM. Finally, the samples were concentrated to a Chl concentration of *c*. 1.5–2 mg ml^−1^.

### Purification of PSI core and LHCI

PSI core complex and LHCI were purified from WT and mutant lines as described by Croce *et al*. (1998) and Wientjes & Croce (2011) with some modifications. Briefly, PSI‐LHCI from sucrose gradient ultracentrifugation was diluted in 10 volumes of 5 mM tricine (pH 7.8) and centrifuged for 3 h at 411 000 ***g***, using a T‐865.1 rotor, Sorvall. Pellets were resuspended in a buffer containing 5 mM Tricine (pH 7.8) and 0.05% β‐DM. The Chl concentration was adjusted to 0.3 mg ml^−1^, and the samples were solubilized by adding 1% β‐DM and 0.5% Zwittergent 3–16. The samples were kept on ice with gentle agitation for 25 min, then rapidly frozen in liquid nitrogen, and slowly thawed to enhance the yield of detached LHCI. Solubilized complexes were fractionated by sucrose gradient ultracentrifugation at 485 000 ***g*** at 4°C for 6 h, using a Beckman SW60 Ti rotor.

### Spectroscopy and pigment analysis

Absorption spectra were recorded at room temperature (RT, 22°C) using an SLM‐Aminco DW‐2000 spectrophotometer in a buffer containing 10 mM HEPES pH 7.5 and 0.05% β‐DM for native complexes or 80% acetone buffered with Na_2_CO_3_ for pigment extracts, in standard 1 cm path quartz cuvettes. Low temperature (77 K) absorption spectra were recorded by diluting samples at *c*. 0.6 OD at the Q_Y_ peak in 70% glycerol, 20 mM HEPES, pH 7.5, following the method outlined in Yang *et al*. (2003) in 1‐ml methacrylate disposable cuvettes.

Emission and excitation fluorescence spectra were recorded at cryogenic temperatures (77 K) using a Jobin–Yvon Fluoromax‐3 spectrofluorometer. Samples were diluted at *c*. 0.2 OD at the Q_Y_ peak in a buffer containing 50% w/v glycerol, 10 mM HEPES, pH 7.5, 0.05% β‐DM. One milliliter of samples were frozen into Aldrich ColorSpec NMR tubes and excited at 440 nm.

Emission spectra of intact leaves were recorded at 77 K using an Ocean Insight SR‐6NVN500‐50 spectrofluorometer. Leaves were frozen into a nitrogen bath and directly measured with an optical fiber.

Circular Dichroism (CD) spectra were recorded at 4°C on a Jasco J1500 spectropolarimeter.

The pigment composition of LHCI complexes was assessed from the deconvolution of acetonic spectra as described in Chazaux *et al*. (2022). High‐performance liquid chromatography (HPLC) pigment separation and quantification were performed according to Gilmore & Yamamoto (1991) by using an Agilent 1260 Infinity II HPLC system.

### Cryo‐EM sample preparation and data acquisition

The PSI supercomplex at a Chl concentration of *c*. 1.5–2 mg ml^−1^ was vitrified with a Mark IV Vitrobot (Thermo Fisher Scientific, Waltham, MA, USA). Three microliters of the sample was applied to a Quantifoil R 0.6/1Cu 300‐mesh grid previously glow‐discharged at 30 mA for 30 s in a GloQube (Quorum Technologies, Laughton, East Sussex, UK). Immediately after sample application, the grids were blotted in a chamber at 4°C and 100% humidity and then plunge‐frozen into liquid ethane.

Vitrified grids were transferred to a Talos Arctica (Thermo Fisher Scientific) operated at 200 kV and equipped with a Falcon 3 direct electron detector (Thermo Fisher Scientific). 2716 and 2601 movies were acquired for WT and *a603‐NH* mutant, respectively, at a nominal magnification of 120 000×, corresponding to a pixel size of 0.889 Å/pixel, in electron counting mode, with a nominal defocus range of −0.8 to −2.4 μm and with a total dose of 40 e^−^/Å^2^ equally distributed on 40 frames. The Cryo‐EM experiments were conducted at the NoLimits Center of the University of Milan.

### Cryo‐EM data processing and image reconstruction

All image processing and reconstruction steps were performed using CryoSparc v.4.4 (Punjani *et al*., 2017). The experimental workflows are outlined in Figs S2 and S3. After patch motion correction and Contrast Transfer Function (CTF) estimation, 2313 and 2257 manually curated micrographs were used for initial particle picking for WT and *a603‐NH* mutant, respectively.

For *At*PSI‐WT, after a first round of reference‐free autopicking and 2D classification, the best classes were used for template‐based autopicking, resulting in an initial number of 575 271 particles. Particles were extracted with a box size of 448 px, binned 2 × 2, and subjected to several rounds of 2D classification. The best 2D classes (282 376 particles) were used for ab initio reconstruction (three classes) and heterogeneous refinement, resulting in an initial map at 3.74 Å resolution. After 3D classification, local and global CTF refinements were used to determine per‐particle defocus and to correct for higher order aberrations (beam tilt and trefoil) and anisotropic magnification, respectively. The best particles were re‐extracted at full size and used for a final round of nonuniform refinement (Punjani *et al*., 2020), obtaining a final density map at 3.13 Å resolution.

For *At*PSI‐*a*603‐NH, 531976 particles generated from a first round of reference‐free autopicking were subjected to 2D classification, and the best classes were used to train the neural network in Topaz (Bepler *et al*., 2019) on a subset of 157 micrographs. The trained model was then used to pick the entire dataset, resulting in a total number of 115 589 particles. These particles were extracted with a box size of 448 px, binned 2 × 2, and after several rounds of 2D classification, the best particles were selected for ab initio reconstruction (3 classes) and heterogeneous refinement, resulting in an initial 4.19 Å resolution map. After two rounds of 3D classification, particles corresponding to the best 3D class were re‐extracted without downscaling and, following local and global CTF refinement as described above, further subjected to homogeneous and nonuniform refinement, yielding a final map with a resolution of 3.29 Å.

Before model building, the maps were sharpened with a global B factor of 83.7 Å^2^ and 91.8 Å^2^ for WT and *a603‐NH* mutant, respectively. The resolution was estimated by the ‘gold‐standard’ Fourier shell correlation (FSC = 0.143) criterion. Local resolution estimation was performed as implemented in CryoSPARC on the unsharpened map. The final EM maps (colored according to the local resolution) and the FSC curves are shown in Figs S2 and S3 (panels D and E).

### Model building and refinement

The high‐resolution structure of the *P. sativum* PSI‐LHCI complex (Protein Data Bank (PDB) code: 7DKZ) was used as the starting model. After initial docking of the model in the map with UCSF ChimeraX (Goddard *et al*., 2018), each protein chain was independently rigid‐body fitted and refined with Phenix‐refine (Adams *et al*., 2010). The amino acid sequences of the different subunits were manually mutated to match the sequences of *A. thaliana* using COOT (Emsley & Cowtan, 2004), and the resulting model underwent several rounds of real‐space refinement in Phenix and manual rebuilding in COOT. Ligands and water molecules were modelled when unambiguously identified in the density map and refined to a reasonable B factor. Ligand restraints for refinement were generated with eLBOW (Moriarty *et al*., 2009). The stereochemical quality of the final model was assessed with MolProbity (Chen *et al*., 2010). Data collection and model refinement statistics are summarized in Table S3. High‐resolution figures were prepared with ChimeraX and PyMol (The PyMOL Molecular Graphics System, v.2.0 Schrödinger, LLC).

### Energy transfer and excitonic coupling calculations

We used a point‐dipole approximation (PDA) to compute the excitonic couplings (EC), as described by van Amerongen & van Grondelle (2001), Müh *et al*. (2010), Liguori *et al*. (2015), and Sen *et al*. (2021). The Chl point dipoles were positioned at the geometric centers of the four nitrogen atoms of the chlorin ring (Friedl *et al*., 2022) $R=\frac{1}{4}\left(R^{\mathrm{NA}}+R^{\mathrm{NB}}+R^{\mathrm{NC}}+R^{\mathrm{ND}}\right)$.

The Chl Q_Y_ and Q_X_ transition dipole moments were aligned parallel to the nitrogen ND‐NB and NC‐NA axis, respectively, as in van Amerongen & van Grondelle (2001); Georgakopoulou *et al*. (2007); Liguori *et al*. (2015). The carotenoids (Car) point dipole was placed on the C15 atom, with transition dipole moments (transition S2 ← S0) oriented parallel to the central part of the polyene chain (atoms C11–C33), as modelled in previous studies (Georgakopoulou *et al*., 2007; Liguori *et al*., 2015). The Q_Y_ transition dipole moments were used for couplings between Chls (Chl *a*–Chl *a*, Chl *a*–Chl *b*, and Chl *b*–Chl *b*), while couplings between Chls and Cars were computed with the Q_X_ transition dipole moments for Chls (Croce *et al*., 2001; Polívka & Frank, 2010).

The ECs between two pigments *i* and *j*, in the PDA were calculated using the following formula (cm^−1^):

$$V_{\mathrm{ij}}=\frac{f_{1}^{2}\left|\mu_{i}\right||\mu_{j}|}{\varepsilon_{r}}\cdot 5.04\frac{\kappa_{\mathrm{ij}}}{R_{\mathrm{ij}}^{3}}$$

where *f*_1_ is the local field correction factor, $\left|\mu_{i}\right|$ and $\left|\mu_{j}\right|$ are the module of the transition dipole moment of pigments *i* and *j*, ε_r_ is the relative dielectric constant, here equal to 2.4 (van Amerongen & van Grondelle, 2001; Liguori *et al*., 2015) $R_{\mathrm{ij}}$ is the module of the distance between the center of the dipole moment vector, and $\kappa_{\mathrm{ij}}$ is the orientation factor between Chl *i* and *j*

$$k_{\mathrm{ij}}=\left({\hat{\mu }}_{i}\cdot {\hat{\mu }}_{j}\right)-3\cdot \left({\hat{\mu }}_{i}\cdot {\hat{r}}_{\mathrm{ij}}\right)\left({\hat{\mu }}_{j}\cdot {\hat{r}}_{\mathrm{ij}}\right)$$

with ${\hat{\mu }}_{i}$ and ${\hat{\mu }}_{j}$ are the normalized transition dipole moment vector and ${\hat{r}}_{\mathrm{ij}}$ is the normalized distance vector. Dipole moment values were taken as 4 D, 3.4 D, and 4.5 D for Chl *a*, Chl *b*, and Cars, respectively (van Amerongen & van Grondelle, 2001; Georgakopoulou *et al*., 2007; Liguori *et al*., 2015). In the case of ε_r_ = 2.4, (*f*_1_^2^μ^2^)/ε_r_ was calculated to be 17.6 D^2^ for Chl *a* (Gobets & Van Grondelle, 2001). The calculations were run via homebuilt codes using Python 3.8.

### Excited state calculations

We performed quantum mechanics/molecular mechanic (QM/MM) optimizations and polarizable QM/MM (Bondanza *et al*., 2020) excited‐state calculations of the Chls in the Lhca4 structure from the present work. Before QM/MM calculations, the structure was refined in a pure MM protocol as detailed in the Supporting Information. All Chls were optimized independently, except for *a*603–*a*609, which were optimized together. Excited‐state calculations were performed for all Chls in Lhca4 and for the *a*603–*a*609 dimer; control calculations were performed by also including L2 Vio with Chls *a*603 and *a*609. We employed a diabatization procedure to extract CT energies and couplings from the dimer calculations (Nottoli *et al*., 2018). Finally, we built an exciton model considering all Q_Y_ states of Chls and the CT states within the *a*603–*a*609 dimer, which we used to simulate absorption spectra of Lhca4 (Sláma *et al*., 2023). Detailed computational methods are reported in the SI (Methods S1).

### Statistics

Statistical analyses were performed in OriginPro using one‐way analysis of variance (ANOVA); means were separated with Tukey's *post hoc* test at a significant level of *P* < 0.05 (see figure legends for details). Error bars represent the SD.

## Results

The *koLhca3 koLhca4 A. thaliana* genotype was complemented with WT or mutant isoforms of Lhca3 and Lhca4, in which the Asn responsible for Chl *a*603 binding was replaced with a His (*a*603‐NH mutant) in both antenna subunits (Asn99 in AtLhca4 and Asn103 in AtLhca3). In the leaves of plants transformed with the *a*603‐NH mutation, we observed a shift in the 77 K fluorescence emission spectrum from 736 to 724 nm (Fig. S4).

**Fig. 2:**
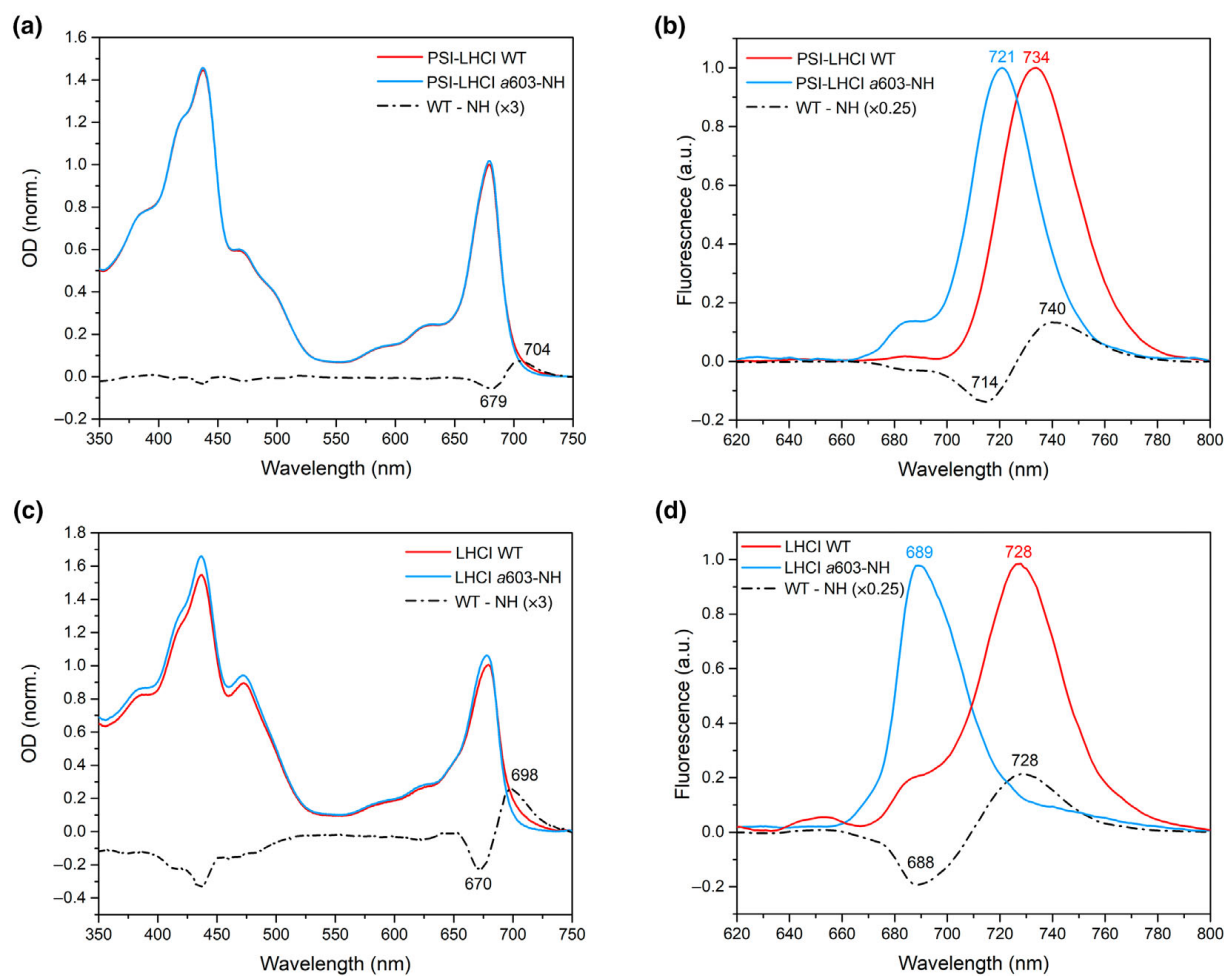
Spectral analysis of PSI‐LHCI and LHCI from *Arabidopsis thaliana* wild‐type (WT) and *a*603‐NH (a, c) Room temperature (RT) absorption and (b, d) 77 K fluorescence emission spectra of PSI‐LHCI (exc. λ = 440 ± 5 nm) (a, b) and LHCI (c, d) from *A. thaliana* WT and *a*603‐NH. The WT minus NH difference spectra are shown as black lines in the corresponding plots. The amplitude of the absorption difference spectra was magnified by a factor of 3, while the fluorescence difference spectra were multiplied by a factor of 0.25 in order to plot them on the same axis. Key wavelengths are indicated in nm above the respective peaks. Experiments were repeated twice independently, with similar results. PS, photosystem; LHC, light harvesting complex.

PSI‐LHCI supercomplexes from both WT and *a*603‐NH mutant lines were purified by sucrose gradient ultracentrifugation (Fig. S1). RT absorption spectra confirmed the loss of far‐red spectral forms in the *a*603‐NH mutant complex, which peaked at 704 nm and extended to 750 nm. This was accompanied by an increased absorption in the 650–700 nm region (the negative peak at 679 nm visible in the difference spectrum; Fig. 2a). Low temperature (77 K) emission spectra revealed a 13‐nm blue shift in the *a*603‐NH mutant PSI supercomplexes compared with WT, its emission peaking at 721 nm (Fig. 2b). Emissions *c*. 720 nm can be attributed to the PSI core complex (Bassi & Simpson, 1987), leading us to conclude that the *a*603‐NH mutation effectively abolished Lhca‐associated RF *in vivo*, consistent with previous reports (Morosinotto *et al*., 2003; Li *et al*., 2023). No change in emission peak was observed in the complex purified from ko plants complemented with WT Lhca3 and Lhca4 (*A3WT‐A4WT*; Fig. S5a,b). The introduction of the *a*603‐NH mutation in either Lhca3 (*A3NH‐A4WT*) or Lhca4 (*A3WT‐A4NH*) induced spectral properties intermediate between WT and *a*603‐NH genotypes (Fig. S5c–e). Although the absorption of the PSI‐LHCI complex in the far‐red region (700–750 nm) was the same in the two single mutants (Fig. S5d), the low‐temperature emission of *A3WT‐A4NH* was slightly shifted toward blue (731 vs 733 nm; Fig. S5e), thus confirming that Lhca4 is the most red‐shifted LHCI, as previously reported (Morosinotto *et al*., 2003; Li *et al*., 2023).

To assess the impact of the *a*603‐NH mutation on the spectral properties of LHCI complexes compared with PSI‐LHCI supercomplexes, we purified LHCI heterodimers (Lhca1–Lhca4 and Lhca2–Lhca3) from WT and *a*603‐NH mutant. RT absorption spectra of LHCI complexes purified from the *a*603‐NH mutant showed the loss of a broad absorption tail, covering the range from 680 to 730 nm and peaking at 698 nm (Fig. 2c). The 77 K emission maximum of LHCI WT dimers was recorded at 728 nm, consistent with (Wientjes & Croce, 2011). By contrast, LHCI dimers from the *a*603‐NH line showed an emission peak at 689 nm, highlighting a significant blue shift of *c*. 39 nm (Fig. 2d), which aligns with observations made *in vitro* (Morosinotto *et al*., 2003). Conversely, the PSI core purified from both WT and *a*603‐NH lines exhibited unchanged optical properties, maintaining a peak at *c*. 720 nm (Fig. S6).

### Structures of PSI‐LHCI from WT and *a*603‐NH plants

To investigate the structural determinants underlying the blue‐shifted absorption/emission caused by the *a*603‐NH mutation, we determined the Cryo‐EM structures of the *A. thaliana* PSI‐LHCI WT and *a*603‐NH supercomplexes. The final reconstructions, at 3.13 Å and 3.29 Å resolution, respectively, revealed well‐defined density maps for both the PSI core and the LHC subunits (Fig. S7), allowing for the construction of accurate models for the two complexes.

The structure of the individual subunits and the positioning of the chromophores within the *At*PSI‐LHCI WT supercomplex were very similar to those observed in other land plants (Qin *et al*., 2015; Mazor *et al*., 2017; Iwai *et al*., 2024; Nelson, 2024; Figs S8–S13, Table S1). In both *At*PSI‐LHCI WT and *a*603‐NH structures, the primary difference in pigment composition between the different Lhcas was found in the Chl*a* : Chl*b* ratio (Fig. S14). Specifically, Lhca1/a2/a3 each bound 14 chl, while Lhca4 bound 15 Chls.

Chl *a*615 was coordinated by His168 and His151 from helix C in Lhca3 and Lhca4, respectively. These pigments showed clear density maps in both structures, allowing for accurate modelling of the Chls (including the chlorin rings and parts of the phytol tails) and the Vio molecule (Fig. S15). An additional Xan molecule (Lut) was located close to Chl *a*615 in Lhca4 only (Fig. S14), as previously observed also in the pea PSI‐LHCI structure (Qin *et al*., 2015). Since this Lut was absent in the red‐emitting subunit Lhca3, it was not considered a potential component of the red‐emitting cluster.

**Fig. 3:**
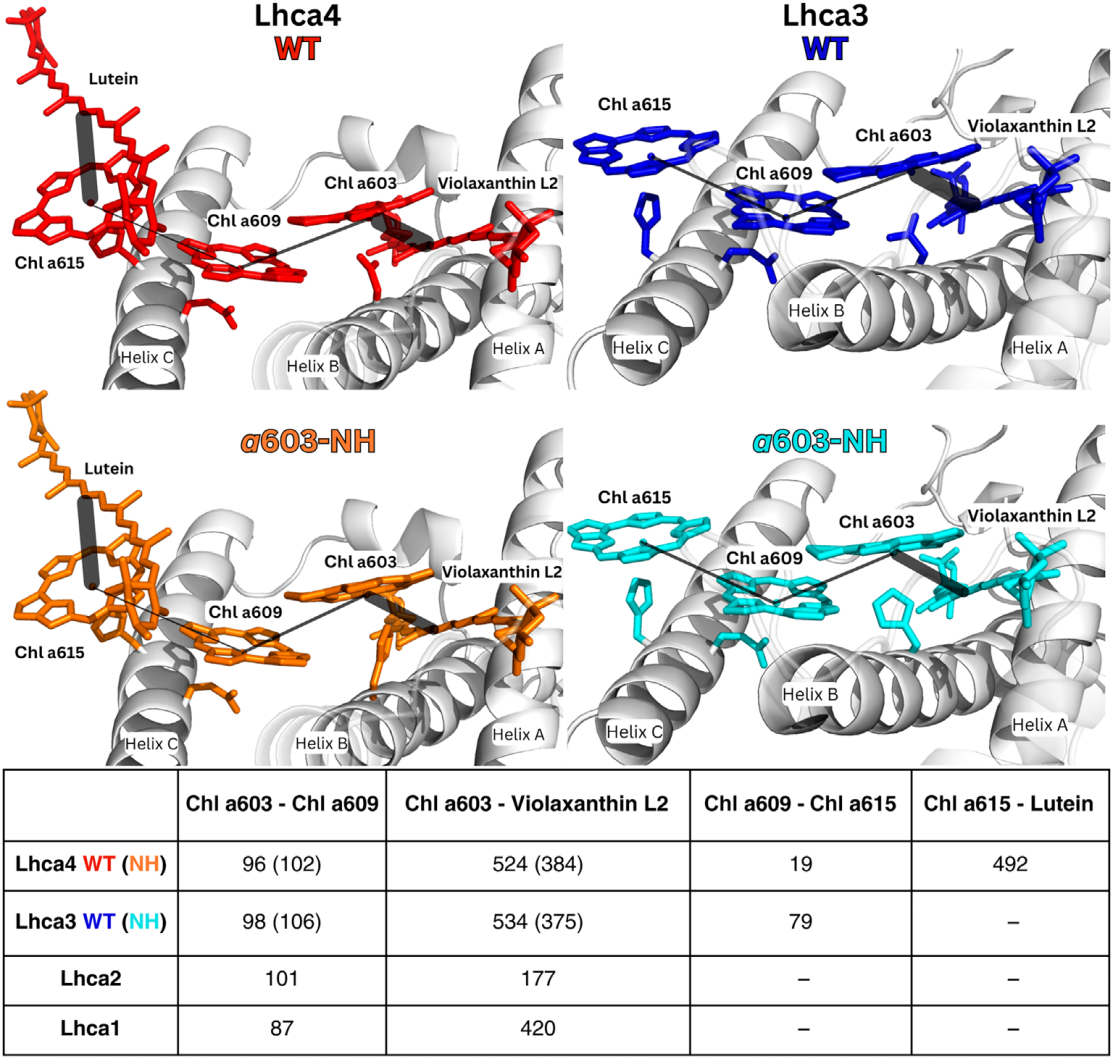
Structural superposition and excitonic coupling analysis of Lhca4 and Lhca3 wild‐type (WT) and *a*603*‐NH*. (*upper part*) Structures of WT and *a*603‐NH red clusters of Lhca4 and Lhca3 from *Arabidopsis thaliana* PSI‐LHCI. WT structures are colored in red and orange, while *a*603‐NH mutant structures are colored in blue and cyan. Lines are drawn between the pigments to highlight the excitonic couplings (EC) reported in the table (*lower part*, values in cm^−1^). The thickness of the line is proportional to the EC value.

In the *a*603‐NH mutant structure, the bulkier His side chain at position 103 (Lhca3) and 99 (Lhca4) caused a *c*. 0.7 Å displacement of the Chl *a*603 chlorin ring (Figs 3, S15–S18), clearly visible in the corresponding density maps (Fig. S15). The chlorin ring and part of the phytol tail of Chl *a*603 were well defined in both the WT and *a*603‐NH density maps, allowing the reliable assignment of the position of Chl *a*603 in both structures (Fig. S15b,c). Surprisingly, the distance between the chlorin ring centers of Chl *a*603 and Chl *a*609 remained essentially unchanged across both WT and mutant proteins (9.3 Å in WT vs 9.1 Å in the *a*603‐NH for Lhca3, and 9.3 Å vs 9.2 Å for Lhca4), as well as the distance between the centers of the closest pyrroles of the two Chl (ring C), which changed slightly from 4.2 to 4.4 Å in Lhca3 and from 4.3 to 4.6 Å in Lhca4 (Fig. S16).

At the same time, the distance between Chl *a*603 and Vio in L2 increased from 5.2 Å in WT to 5.9 Å in *a*603‐NH, and from 5.3 Å to 6.0 Å in both Lhca3 and Lhca4, with a change of *c*. 12% in both cases.

We then computed EC values, which account for the relative spatial orientation between each pair of pigments in the cluster. The EC between Chl *a*603 and Vio decreased from 534 cm^−1^ and 524 cm^−1^ for Lhca3 and Lhca4 in the WT to 375 cm^−1^ and 384 cm^−1^ in the *a*603‐NH mutant, representing a change of 27–29% (Figs 3, S19). By contrast, the introduction of the *a*603‐NH mutation resulted in quite small changes in the EC values between the Chl *a*603 and Chl *a*609 pair (6–8 cm^−1^). Furthermore, while the EC values between the Chl pairs were in the limited range of 87 cm^−1^ and 101 cm^−1^ for all Lhca, the EC values for the WT Chl *a*603–Vio L2 coupling were significantly higher for Lhca3 and Lhca4 than for Lhca1 and Lhca2, which do not show red‐shifted absorption. Notably, the *a*603‐NH mutation decreased the ECs of Lhca3 and Lhca4 to values comparable to those of the ‘non‐red’ subunits. Together, this evidence suggests that the Vio ligand might play a role in tuning the absorption properties of Lhca3 and Lhca4 toward low energy levels.

**Fig. 4:**
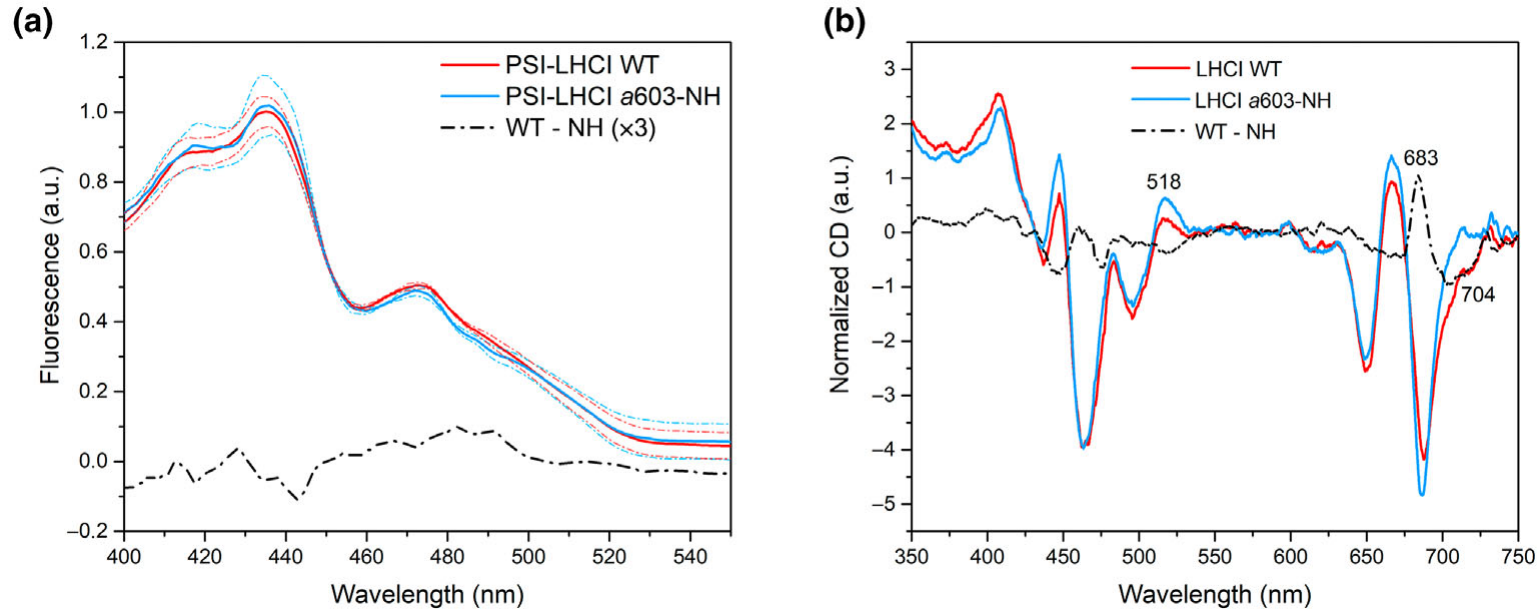
Spectral analysis of PSI‐LHCI and LHCI from *Arabidopsis thaliana* wild‐type (WT) and *a*603‐NH. (a) Low‐temperature (77 K) fluorescence excitation spectra of PSI‐LHCI from *A. thaliana* WT and *a*603‐NH. Far‐red fluorescence emission (734 ± 5 nm for WT and 721 ± 5 nm for the *a*603‐NH mutant) was followed by exciting samples from 400 to 550 nm. Spectra were normalized to the integrated area under the curve. (Solid lines represent the average of *n* = 3 independent biological replicates, broken lines represent the SD) The difference spectrum was magnified by a factor of 3 for better visualization. (b) circular dichroism (CD) (4°C) spectra of purified LHCI complexes from WT and *a*603‐NH mutants. Spectra are normalized to the same absorption in the Q_Y_ region. The difference spectra are shown as black lines in the corresponding plots. The experiments were independently repeated twice with similar results. PS, photosystem; LHC, light harvesting complex.

To investigate the role of the Vio at the L2 site of Lhca3 and Lhca4 in modulating RF‐inducing interactions, we compared the 77 K fluorescence excitation spectra of WT and *a*603‐NH PSI‐LHCI, highlighting the contribution of different wavelengths and associated chemical species to their far‐red fluorescence emissions. In the 480–510 nm region, where the largest contribution in absorption comes from Cars (Ashenafi *et al*., 2023), the *a*603‐NH mutant showed only slightly lower signal than WT (Fig. 4a), suggesting a small reduction in energy transfer efficiency from Car to Chls in the mutant (Fig. 4a). The difference between the 77 K absorption and excitation (Abs‐Exc) spectra in Fig. S20(a) provides insight on the efficiency by which the absorbed energy is transferred to the lowest energy emitter at each wavelength. In the 480–510 nm region, the Abs *minus* Exc values for the *a*603‐NH mutant were somewhat larger than those of the WT, suggesting a stronger energy dissipation and a reduced energy transfer toward Chl *a*, consistent with the lower calculated EC value (Fig. 3). We proceed to further investigate the role of Vio in promoting RF. Since the removal of Vio in site L2 is not feasible due to its essential role in protein folding and stability (Dall'Osto *et al*., 2013), we analyzed the effect of altering L2 occupancy on the PSI‐LHCI far‐red absorption. In the *npq2* genotype, zeaxanthin (Zea) replaces Vio in all sites due to zeaxanthin epoxidase inactivation (Niyogi *et al*., 1998; Ballottari *et al*., 2014). 77 K fluorescence emission spectra showed a slight, *c*. 2 nm, blue shift in *npq2* PSI‐LHCI vs WT (Fig. S21a). Notably, the Vio → Zea exchange also led to an apparent decrease in Chl a/b ratio in the PSI‐LHCI supercomplex, reflected in enhanced absorption between 462 and 492 nm, consistent with previous findings (Ballottari *et al*., 2014), which, however, was not conserved upon purification of the LHCI dimers (Fig. S21c,d). Pigment composition analysis of LHCI also revealed an altered Chl/Car ratio in the *npq2* line, with a significant decrease in Lut content with respect to both WT and *a*603‐NH. This was partially compensated by the accumulation of Zea. In this view, the small spectral differences observed in *npq2* compared with WT could also stem from secondary factors, such as altered carotenoid profiles (Fig. S21).

To analyze pigment‐pigment interaction in WT and *a*603‐NH LHCI complexes, we recorded CD spectra in the visible region (350–750 nm). In the Q_Y_ region, the spectra of both WT and *a*603‐NH line displayed signature peaks typical of LHC (i.e. −/+/−), indicating a similar and conserved structural conformation and pigment organization (Mozzo *et al*., 2008). The CD spectra of the WT and *a*603‐NH complexes revealed the largest difference in the far‐red region (λ > 700 nm), where the mutant showed a markedly reduced (−) signal associated with the excitonic interaction responsible for the RF (Fig. 4b). The difference spectrum (black line) evidenced the disappearance of a (−) low‐energy band in *a*603‐NH LHCI (690–730 nm), which was compensated by a high‐energy (+) band appearing at 683 nm. This conservative signal aligns with the loss of excitonic interactions between Chl *a*603 and *a*609, as previously reported (Morosinotto *et al*., 2003; Wientjes *et al*., 2012). In the Soret region, the interpretation of the CD spectra is complicated by the superimposition of signals from Chls and Cars. Minor differences are also present between 465 and 530 nm when comparing the *a*603‐NH with the WT. These differences, however, can be attributed to actual changes in pigment‐pigment interactions as well as to small losses of Cars during purification (Fig. S21d).

Overall, our results suggest that the Vio in L2, despite strong EC with Chl *a*603 in Lhca3 and Lhca4, has only a modest influence on the spectral properties and red‐light absorption of the *a*603–*a*609 pair.

### Excited state calculations

**Fig. 5:**
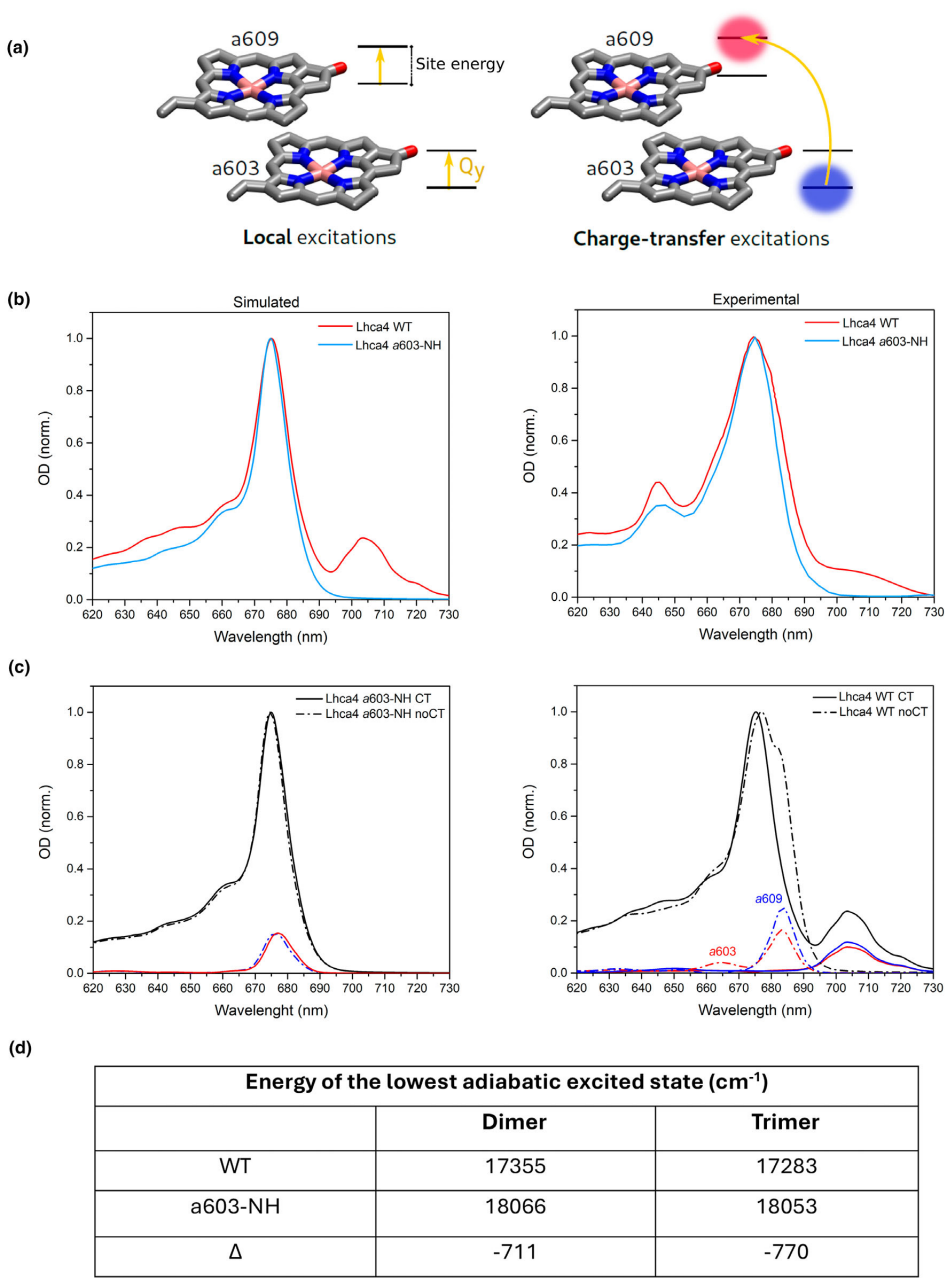
Role of charge‐transfers (CTs) in enhancing far‐red absorption. (a) Schematic depiction of local excitations (left) and charge transfer excitations (right). In local excitations, electron transitions occur within orbitals of the same molecule; in charge‐transfer excitations, electrons are promoted from orbitals localized on one molecule to orbitals localized on another molecule. (b) Comparison of wild‐type (WT) and *a*603‐NH absorption spectra in the simulations (left) and experiments for isolated Lhca4 (right). (c) Effect of adding CT excitations to the exciton model in the *a*603‐NH mutant (left) and WT (right). In (b, c) the simulated spectra were rigidly shifted by −1800 cm^−1^ to account for time‐dependent density functional theory (TD‐DFT) systematic error. (d) Energy of the lowest excited state in the *a*603–*a*609 dimer and in the *a*603–*a*609–Vio trimer. The experimental spectra of WT and *a*603‐NH are adapted with permission from Wientjes *et al*. (2012). Copyright © 2012 Elsevier B.V. All rights reserved.

To model the effect of the *a*603‐NH mutation on the red‐shifted absorption at the molecular level, we performed structure‐based polarizable QM/MM calculations of excited states on the Lhca4 WT and *a*603‐NH mutant models, including charge‐transfer excitations (Fig. 5a). In the spectra simulated with the standard exciton model (noCT in Figs 5c, S22), neither the WT nor the *a*603‐NH exhibited a signature of the RF observed in the experiments, notably the broad band peaking at > 700 nm. Conversely, by adding the CT contribution for the *a*603–*a*609 dimer in the calculations, we observed a low energy band (*c*. 710 nm) in the simulated spectrum of the WT (Fig. 5b), resembling the experimental observations in the isolated rLhca4 (Wientjes *et al*., 2012). By contrast, virtually no changes in the spectrum were observed for the mutant (Fig. 5c). This indicates that the major contribution to the WT/*a*603‐NH absorption shift resides in the different extent of the coupling of CT states to local excitations between Chl *a*603–*a*609, supporting a previous proposal (Wientjes *et al*., 2012; Sláma *et al*., 2023). CT has a similar effect on the energy of the exciton state regardless of the CT direction; therefore, we included both CT states (*a*603 + *a*609− and *a*603–*a*609+) in our calculations. It turned out that both CT states are coupled to the Q_Y_ states as found previously (Sláma *et al*., 2023), but the *a*603 + *a*609− is significantly lower in energy than the opposite state. We can therefore conclude that the lowest exciton state of WT Lhca4, responsible for the red forms, is a mixture of exciton and CT states, with a larger component of the *a*603 → *a*609 CT (Table S4).

A secondary effect can be noticed in the WT spectrum simulated without CT states (Fig. 5c), namely the appearance of a red‐shifted shoulder, corresponding to *a*603–*a*609 absorption. While this shoulder is clearly more blue‐shifted than the red band at *c*. 710 nm, it indicates red‐shifted site energies for *a*603–*a*609, in contrast with the *a*603‐NH mutant. Nonetheless, the model predicts the significantly red‐shifted band observed in the experiment only when including CT states.

We also assessed the electronic interactions between the Chl dimer and L2 Vio. To this end, we computed the excited states for two supermolecules, one formed by *a*603 and *a*609 (dimer), and the other formed by the same two Chls and the Vio (trimer). We investigated the energy of the lowest excited state responsible for fluorescence emission, to explore the effect on the electronic structure of including or excluding Vio (Fig. 5d). A change by *c*. 15 and 70 cm^−1^ was observed when comparing the dimer to the trimer supermolecule, for both *a*603‐NH and WT, respectively, suggesting a marginal involvement of the Vio in L2 in tuning the low‐energy absorption of the cluster, consistent with our experimental results (Fig. 4). However, part of this difference is already explained by the varying treatment of the Vio (as point charges in the dimer vs an active molecule in the trimer). Therefore, such a difference is not large enough to conclude that Vio in L2 effectively mixes its orbitals with the two Chls or contributes to the CT excitation within the *a*603–*a*609 pair (Fig. S27). We conclude that the difference between the WT and the *a*603‐NH species (Δ) was not significantly changed by the inclusion of Vio.

### The role of the extra chromophore Chl *a*615

Lhca3 and Lhca4 bound an additional Chl molecule, *a*615 (referred to as Chl *a*617 in Qin *et al*., 2015), which was coordinated by a His residue located on either the third or fourth turn of the C helix of Lhca3 and Lhca4, respectively (Fig. 3). This pigment was absent in the ‘blue’ subunits Lhca1 and Lhca2, leading to speculation about its involvement in RF formation (Melkozernov & Blankenship, 2003; Fig. 3), since the positioning of Chl *a*615 allowed favorable dipolar coupling with Chl *a*609. Notably, the EC value between Chl *a*615 and Chl *a*609 was 79 cm^−1^ for Lhca3, whereas it dropped to 19 cm^−1^ in Lhca4 due to the differing orientation of the chlorin ring in relation to Chl *a*609. Although His residues were also present in the second turn of the C helix of Lhca1 and Lhca2, the lack of electronic density from Cryo‐EM suggested that Chl *a*615 was absent in these subunits.

An additional Lut molecule was located near the chlorin ring of Chl *a*615 in Lhca4 (Fig. S23a), positioned to allow strong dipolar coupling (492 cm^−1^) with this Chl. This Lut had previously been observed only in the Cryo‐EM structure of *Zea mays* PSI‐LHCI (PDB 5ZJI; Pan *et al*., 2018) and in the X‐ray structure of *P. sativum* PSI‐LHCI (PDB 4XK8; Qin *et al*., 2015). The Lut was situated at the interface between Lhca1 and Lhca4, in contact with both subunits, and one of its hydroxyl groups formed a hydrogen bond with the carbonyl oxygen of Ser210 in Lhca1 (Fig. S23b). Consequently, it might get lost during the purification of monomeric Lhca4.

To investigate the potential role of Chl *a*615 in far‐red light absorption, we generated mutants lacking this chromophore in both Lhca3 and Lhca4 by substituting the His‐binding residue with nonbinding Ala (*a*615‐H → A) or Ile (*a*615‐H → I; Fig. S24; Remelli *et al*., 1999; Guardini *et al*., 2022). Immunoblotting analysis of thylakoid membranes from the *a*615‐HA and *a*615‐HI mutant lines revealed a slight reduction in Lhca3 and Lhca4 protein levels (Fig. S26a,b). While this reduction was not statistically significant for Lhca3, it likely reflects a general decrease in the stability of Lhca complexes lacking Chl *a*615, rather than a transcriptional effect. This interpretation is supported by the comparable accumulation of Lhca proteins in *A3WT–A4WT* lines and WT *Arabidopsis*, and is consistent with previous *in vivo* observations of other Chl‐deficient LHC complexes (Guardini *et al*., 2020).

**Fig. 6:**
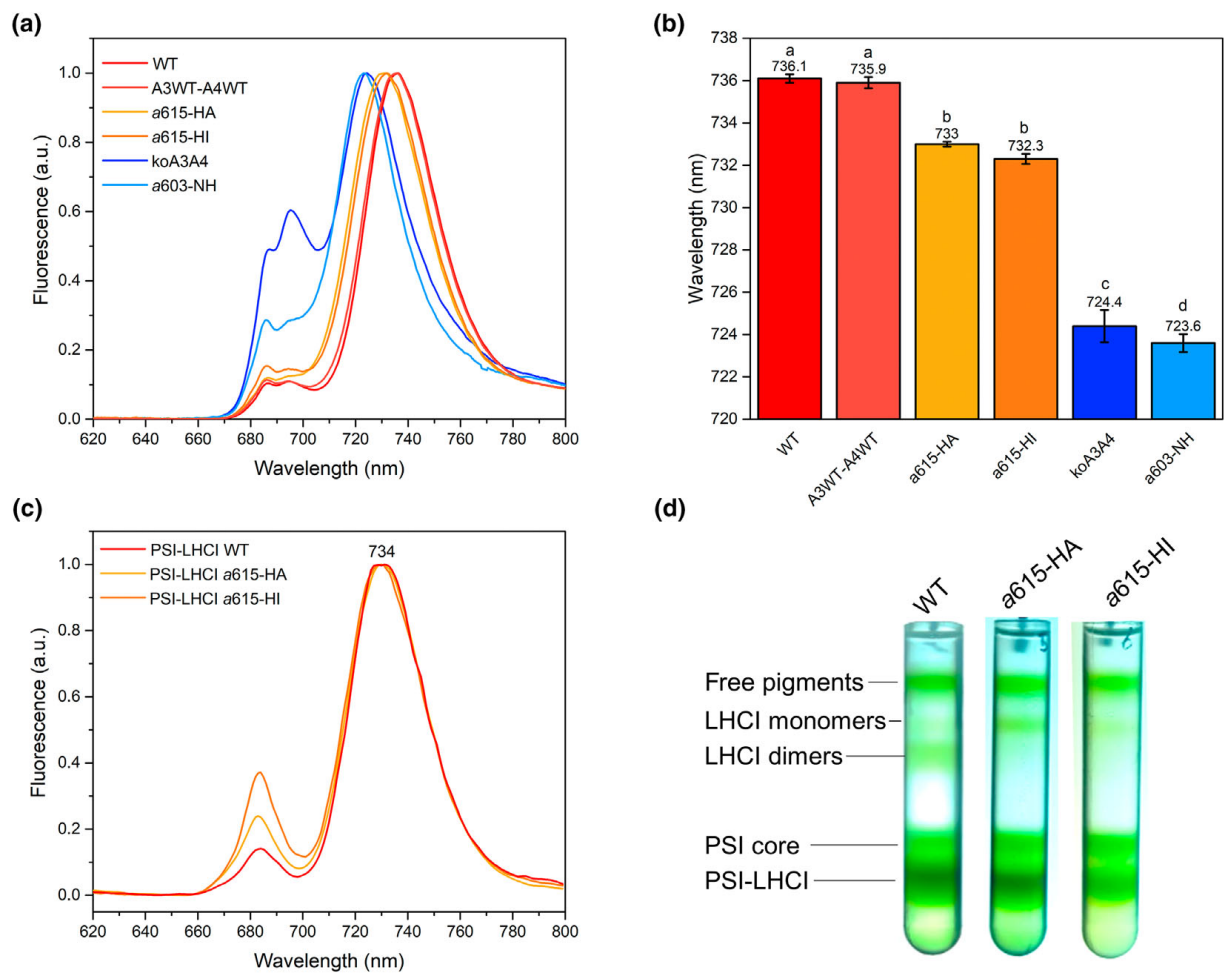
Spectral characteristic of different Chl‐binding mutants of *Arabidopsis thaliana*. (a) Fluorescence emission spectra measured on frozen intact leaves for different genotypes of *A. thaliana*: wild‐type (WT), *koLhca3 koLhca4* lines complemented with WT sequences of Lhca3 and Lhca4 (*A3WT‐A4WT*), mutants lacking Chl *a*615 (a615‐HA and a615‐HI), *koLhca3 koLhca4* (*koA3A4*), and *a*603‐NH mutant (a603‐NH), and normalized to the λ_max_. (b) Barplot of the peak emission wavelength (λ_max_), measured on the same genotypes. The values on the individual bars represent the mean λ_max_ in nm; the error bars correspond to the SD (*n* ≥ 5 independent biological samples). Values that are significantly different (ANOVA followed by Tukey's *post hoc* test at a significance level of *P* < 0.05) are marked with different letters. (c) PSI‐LHCI 77 K fluorescence emission spectra measured on the WT, *a*615‐HA, and *a*615‐HI genotypes (exc λ = 440 ± 5 nm). (d) Sucrose gradient fractionation of solubilized PSI‐LHCI from WT, *a*615‐HA, and *a*615‐HI plants. Five pigment‐containing bands were resolved and identified as: free‐pigments, monomeric LHCI, dimeric LHCI, PSI core complex, and PSI‐LHCI supercomplex. Experiments in panels (c) and (d) were repeated twice independently, with similar results. PS, photosystem; LHC, light harvesting complex.

When recording 77 K emission spectra from intact leaves of genotypes with and without Chl *a*615, we observed a small blue‐shift from 736 nm to 733 ± 1 nm (Fig. 6a,b). However, in the isolated PSI‐LHCI complex, both genotypes showed the same emission at 734 nm (Fig. 6c). The *c*. 2–3 nm red‐shift in leaf samples compared with isolated supercomplexes can be attributed to self‐absorption effects (Weis, 1985). The *a*603‐NH mutant and *koLhca3 koLhca4* exhibited a blue‐shifted λ_max_ to *c*. 724 nm, similar to the emission of the PSI core complex (Croce *et al*., 1998). We conclude that Chl *a*615 does not play a role in forming RF. To explain the 3 nm shift observed in leaf emission with and without Chl *a*615, we analyzed the pigment‐protein organization of thylakoids by sucrose gradient ultracentrifugation. Fig. S25 compares the fractionation patterns from solubilized thylakoids from the WT and Chl *a*615‐less mutants. In addition to the lowest (higher MW) band containing the fully assembled PSI‐LHCI supercomplex, a prominent green band containing the PSI core complex was present in both *a*615 mutant lines, while it was faint in WT (Wientjes *et al*., 2009). Consistently, the upper band (with the lowest MW) was enriched in the mutants compared with the WT. We interpreted these results as indicating a de‐stabilization of the dimeric Lhca1‐Lhca4 and Lhca2‐Lhca3 dimers consequent to the missing Chl *a*615. This was further confirmed by a second sucrose gradient fractionation upon treating the isolated PSI‐LHCI with Zwittergent 3–16, a procedure that allows the isolation of LHCI dimers from the PSI core (Figs 6d, S26). The pattern from the WT yielded both monomers and dimers of Lhcas, while only monomers were observed in both Chl *a*615 mutant lines. It is worth noting that Chl *a*615 was localized at the interface between Lhca subunits in both Lhca2‐Lhca3 and Lhca1‐Lhca4 subunits (Fig. S24), consistent with the hypothesis of its role in the stabilization of LHCI dimers and the binding to PSI core complex.

## Discussion

**Fig. 7:**
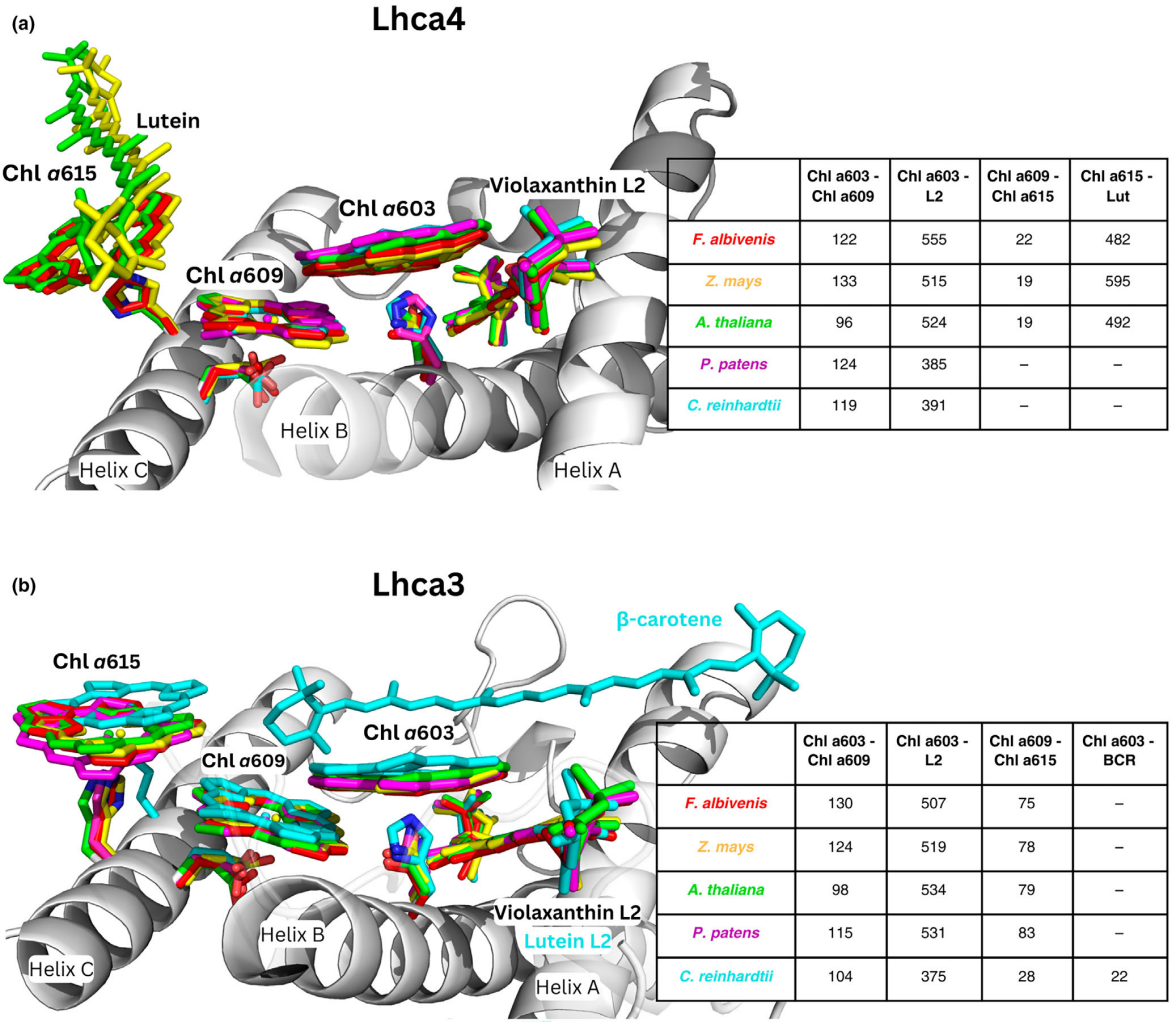
Comparative structural and excitonic coupling analysis of red‐shifted pigments across species. Structural superposition and excitonic coupling analysis (values in cm^−1^) of the red cluster pigments from *Fittonia albivenis* (PDB 8WGH), *Zea mays* (PDB 5ZJI), *Arabidopsis thaliana*, *Physcomitrium patens* (PDB 7XQP), and *Chlamydomonas reinhardtii* (PDB 7ZQC). The analysis focused on (a) Lhca4 or the corresponding subunit, that is Lhca8 for *C. reinhardtii* and Lhca2b for *P. patens* (Yan *et al*., 2021; Gorski *et al*., 2022) and (b) Lhca3.

A key evolutionary trend in the green lineage is the enhancement of far‐red absorption in PSI‐LHCI, associated with the appearance of RFs. These adaptations likely emerged *c*. 489–403 m in response to light filtering under dense vegetation canopies (Fig. 1). Despite clear differences in spectral traits across taxa, the structural similarity of LHCI subunits (Iwai *et al*., 2024; Fig. 7) raises questions about the molecular determinants of far‐red absorption. The presence of an Asn as a ligand for the Chl *a*603 has been reported as required for red‐shifted states (Morosinotto *et al*., 2003; Wientjes *et al*., 2012; Li *et al*., 2023). However, this correlation proves overly simplistic. Indeed, in higher plants which exhibit the strongest far‐red emission (i.e. *A. thaliana*, *Z. mays*, and *F. albivenis*), Chl *a*603 is coordinated by Asn residues in both Lhca3 and Lhca4 (Pan *et al*., 2018; Li *et al*., 2024) while in *C. reinhardtii*, Chl *a*603 is coordinated by a His residue in Lhca3 and Lhca8 (the Lhca4 homolog; Naschberger *et al*., 2022). Furthermore, Asn appears in loosely bound Lhcas (Stauber *et al*., 2009; Su *et al*., 2019), which are located in more distal positions relative to the PSI core complex (Huang *et al*., 2021). In *P. patens*, which occupies an intermediate position both evolutionarily and in terms of absorption properties, an Asn residue coordinates Chl *a*603 only in Lhca3 (Gorski *et al*., 2022). Additionally, seagrasses have retained the Asn ligand while losing RF, suggesting that *a*603 coordination alone is insufficient to explain RF (Fig. 1b,c).

Our PSI‐LHCI *a*603‐NH mutant showed a *c*. 13 nm fluorescence blue shift at 77 K. Upon isolation of LHCI, we observed a *c*. 39 nm emission shift, confirming the direct effect of the Asn → His substitution on the antenna itself (Fig. 2). Cryo‐EM structures revealed a *c*. 0.7 Å repositioning of the *a*603 chlorin ring in the a603‐NH mutant (Fig. S16), weakening coupling with neighboring pigments, particularly Vio in site L2 (Fig. 3). While EC calculations suggested a possible role for Vio L2 in the tuning of the excitonic interaction originating RFs, the QM/MM excited‐state simulations revealed that the low‐energy absorption is primarily due to CT states between *a*603 and *a*609 (Wientjes *et al*., 2012; Sláma *et al*., 2023; Fig. 5). Although L2 Vio does not participate directly (quantum‐mechanically) in the lowest exciton state (Figs 3, 5), it seems to be important in influencing local pigment geometry, and its EC value with Chl *a*603 could still be an indicator to predict the presence of RFs (Fig. 7).

Additionally, Chl *a*615, close to the red cluster (Melkozernov & Blankenship, 2003; Qin *et al*., 2015), showed strong coupling with *a*609 (Fig. 3) but its removal did not affect far‐red emission (Fig. 6c); rather, it appears to play a structural role in LHCI dimers stabilization and in their association with PSI core (Figs 6d, S23b).

In conclusion, our integrated structural, spectroscopic, and computational analyses support a model where CT interactions between *a*603 and *a*609 are the primary drivers of far‐red spectral features (Sláma *et al*., 2023). Other pigments and protein environments modulate, but do not determine, the presence of RF.

Further research will be necessary to better clarify the contribution of the protein environment surrounding the ‘red cluster’, for example, by comparing high‐resolution structures of red‐shifted and blue‐shifted Lhcas, such as from *F. albivenis* (Li *et al*., 2024) and seagrasses.

Understanding these structure–function relationships is essential for future efforts aimed at fine‐tuning Chl absorption toward underutilized spectral regions, an approach with strong potential to enhance photosynthetic efficiency under real field conditions (Ort *et al*., 2015; Cutolo *et al*., 2023).

## Competing interests

None declared.

## Author contributions

RB, SC and LD'O conceived the work and designed the experiments. ZG, VFP and AA carried out the construction of mutants and performed their biochemical and spectroscopical characterization. ZG, SC and AA carried out the preparation of the samples for Cryo‐EM. DMVB and AC‐S conducted the Cryo‐EM sample preparation and data collection. SC and DM analyzed the Cryo‐EM data and reconstructed the PSI‐LHCI structures. DM performed the bioinformatics analysis and EC calculations. EB, CJ, LP‐G, LC and BM carried out the quantum chemical calculations and analyses. DM, SC and ZG wrote the original text draft. SC, ZG, DM and VFP contributed equally to this work. All authors discussed the results and contributed to drafting the manuscript.

## Disclaimer

The New Phytologist Foundation remains neutral with regard to jurisdictional claims in maps and in any institutional affiliations.

## Supporting information


**Fig. S1** Sample preparation and characterization.
**Fig. S2** Cryo‐EM workflow, images and map quality for *At*PSI‐LHCI WT.
**Fig. S3** Cryo‐EM workflow, images and map quality for *At*PSI‐LHCI *a*603‐NH.
**Fig. S4** Emission spectra of leaves of *Arabidopsis thaliana* WT and *a*603‐NH mutant.
**Fig. S5** Characterization of PSI‐LHCI purified from WT, *A3WT‐A4WT*, *A3NH‐A4WT* and *A3WT‐A4NH* mutant lines.
**Fig. S6** Characterization of solubilized PSI‐LHCI from WT and *a*603‐NH.
**Fig. S7** Atomic models of PSI‐LHCI subunits and selected ligands superimposed on Cryo‐EM maps.
**Fig. S8** Overall architecture of the PSI‐LHCI WT supercomplex.
**Fig. S9** Positions of ligands in the PSI‐LHCI WT of *Arabidopsis thaliana* (PDB 9GBI).
**Fig. S10** Lipid arrangement in PSI‐LHCI supercomplexes.
**Fig. S11** Superposition of the PSI WT from *Arabidopsis thaliana* (PDB 9GBI, white) and from *P. sativum* (PDB 7DKZ).
**Fig. S12** Global superposition RMSD of the *At*PSI‐WT (PDB 9GBI) structure with PSI‐WT structures from PDB 8J7B, 8JZA (both from Cryo‐EM data), and 7DKZ.
**Fig. S13** Chl, xanthophyll and lipid arrangement in PSI‐LHCI WT.
**Fig. S14** Pigment content of the LHCI antenna subunits of *Arabidopsis thaliana* WT (PDB 9GBI).
**Fig. S15** Atomic models of the ‘red cluster’ pigments.
**Fig. S16** Distances between Chl *a*603, Chl *a*609, and Violaxanthin L2 in Lhca3 and Lhca4 subunits of *Arabidopsis thaliana*.
**Fig. S17** Superposition of selected Chls and xanthophylls of the Lhca3/Lhca4 WT and *a*603‐NH.
**Fig. S18** Superposition of selected Chls and xanthophylls of the Lhca1‐4 WT and *a*603‐NH.
**Fig. S19** Excitonic coupling absolute values between pigments in LHCI WT and *a*603‐NH.
**Fig. S20** Difference spectra (absorption – excitation spectra, measured at 77 K in the 400–550 nm region) of PSI‐LHCI from *Arabidopsis thaliana* WT and *a*603‐NH.
**Fig. S21** Spectral analysis and pigment composition of PSI‐LHCI from WT and *npq2*.
**Fig. S22** Site energies and simulated absorption spectra.
**Fig. S23** Structural analysis of the Chl *a*615‐Lutein cluster.
**Fig. S24** Structural diagram of PSI‐LHCI supercomplex, Lhca3 and Lhca4 from WT and the a615‐HI line.
**Fig. S25** Sucrose gradient fractionation of thylakoid membranes of WT and Chl *a*615 mutant lines.
**Fig. S26** Characterization of Chl *a*615 mutant lines.
**Fig. S27** Visual representation of the overlap between LUMO orbitals of Chls *a*603 and *a*609 as computed for the WT and *a*603‐NH structures.
**Fig. S28** Alignment of the sequences (exons and introns) of the synthetic genes.
**Fig. S29** Superposition of Cryo‐EM structures of Lhca4 WT or *a*603‐NH mutant with the optimized models used for QM/MM calculations.
**Methods S1** Additional methods.
**Table S1** *At*PSI‐*a*603‐NH structural model.
**Table S2** List of the primers used to obtain and characterize the *a603‐NH* mutant lines.
**Table S3** Cryo‐EM data collection, refinement, and validation statistics.
**Table S4** Energies and couplings of the Q_Y_ and CT states of WT and *a*603‐NH.
Please note: Wiley is not responsible for the content or functionality of any Supporting Information supplied by the authors. Any queries (other than missing material) should be directed to the *New Phytologist* Central Office.

## Data Availability

Sequence data from this article can be found in the Arabidopsis Genome Initiative under accession nos.: At1g61520 (LHCA3), At3g47470 (LHCA4), and At5g67030 (ZE). The KO lines were obtained in the NASC under stock nos.: N876497 (*koLhca3*) and N679009 (*koLhca4*). Mutant line *npq2* was a kind gift of Prof. K.K. Niyogi (University of California at Berkeley). The Cryo‐EM maps and coordinates have been deposited in the EMDB and wwPDB, respectively: PSI–LHCI WT (Cryo‐EM map, EMD‐51219; consensus refinement map; PDB: 9GBI) and PSI–LHCI *a*603‐NH mutant (Cryo‐EM map, EMD‐51227; PDB: 9GC2).
