# Supplementary material for: Structural determinants for red‐shifted absorption in higher‐plants Photosystem I
Source: New Phytol. 2025 Sep 15;248(5):2331–46. doi: 10.1111/nph.70562 (PMC12589708; doi:10.1111/nph.70562)
Supplement: Supplementary file 1 — Fig. S1 Sample preparation and characterization. Fig. S2 Cryo‐EM workflow, images and map quality for AtPSI‐LHCI WT. Fig. S3 Cryo‐EM workflow, images and map quality for AtPSI‐LHCI a603‐NH. Fig. S4 Emission spectra of leaves of Arabidopsis thaliana WT and a603‐NH mutant. Fig. S5 Characterization of PSI‐LHCI purified from WT, A3WT‐A4WT, A3NH‐A4WT and A3WT‐A4NH mutant lines. Fig. S6 Characterization of solubilized PSI‐LHCI from WT and a603‐NH. Fig. S7 Atomic models of PSI‐LHCI subunits and selected ligands superimposed on Cryo‐EM maps. Fig. S8 Overall architecture of the PSI‐LHCI WT supercomplex. Fig. S9 Positions of ligands in the PSI‐LHCI WT of Arabidopsis thaliana (PDB 9GBI). Fig. S10 Lipid arrangement in PSI‐LHCI supercomplexes. Fig. S11 Superposition of the PSI WT from Arabidopsis thaliana (PDB 9GBI, white) and from P. sativum (PDB 7DKZ). Fig. S12 Global superposition RMSD of the AtPSI‐WT (PDB 9GBI) structure with PSI‐WT structures from PDB 8J7B, 8JZA (both from Cryo‐EM data), and 7DKZ. Fig. S13 Chl, xanthophyll and lipid arrangement in PSI‐LHCI WT. Fig. S14 Pigment content of the LHCI antenna subunits of Arabidopsis thaliana WT (PDB 9GBI). Fig. S15 Atomic models of the ‘red cluster’ pigments. Fig. S16 Distances between Chl a603, Chl a609, and Violaxanthin L2 in Lhca3 and Lhca4 subunits of Arabidopsis thaliana. Fig. S17 Superposition of selected Chls and xanthophylls of the Lhca3/Lhca4 WT and a603‐NH. Fig. S18 Superposition of selected Chls and xanthophylls of the Lhca1‐4 WT and a603‐NH. Fig. S19 Excitonic coupling absolute values between pigments in LHCI WT and a603‐NH. Fig. S20 Difference spectra (absorption – excitation spectra, measured at 77 K in the 400–550 nm region) of PSI‐LHCI from Arabidopsis thaliana WT and a603‐NH. Fig. S21 Spectral analysis and pigment composition of PSI‐LHCI from WT and npq2. Fig. S22 Site energies and simulated absorption spectra. Fig. S23 Structural analysis of the Chl a615‐Lutein cluster. Fig. S24 Structural diagram of PSI‐LHCI s [file NPH-248-2331-s001.pdf]

New Phytologist Supporting Information

Structural determinants for red-shifted absorption in higher-plants Photosystem I.

Stefano Capaldi, Zeno Guardini, Daniele Montepietra, Vittorio Flavio Pagliuca, Antonello Amelii, Elena Betti, Chris John, Laura Pedraza-González, Lorenzo Cupellini, Benedetta Mennucci, Diane Marie Valerie Bonnet, Antonio Chaves-Sanjuan, Luca Dall'Osto and Roberto Bassi

Article acceptance date: 19 August 2025

Additional methods:

Assembly of complementation constructs

To generate the *a603-NH* mutant, *LHCA3* and *LHCA4* genes were PCR-amplified from *Arabidopsis* WT (col-0) genomic DNA. The amplified fragments, which included the 5'-UTR and 3'-UTR regulatory regions along with the coding region of *LHCA3* or *LHCA4*, were obtained using the following primer pairs:

Lhca3_FW 5'-GGGGACAAGTTTGTACAAAAAAGCAGGCTTCTGAAGCAGATACTAATCTAATCTGAGC-3';

Lhca3_RV 5'-GGGGACCACTTTGTACAAGAAAGCTGGGTCAAGGTGGTTTGGGAGATACAGA-3'

Lhca4_FW 5'-GGGGACAAGTTTGTACAAAAAAGCAGGCTTCTTTTGCATGGTGATTTCCAG-3'

Lhca4_RV 5'-GGGGACCACTTTGTACAAGAAAGCTGGGTCTCCTCAATCAGGTTGGCTTA-3'

These primers were designed to include attB clamps for the Gateway cloning system (Invitrogen). The resulting amplicons were cloned into pDONR221 vectors and subsequently recombined into pK7WG2 and pH7WG2 plant destination vectors (Karimi et al., 2002) for *LHCA3* and *LHCA4*, respectively. Specific primers (Table **S2**) and the QuickChange Stratagene kit were used to substitute Asn encoding triplets with His triplets. The resulting pK7WG2-*LHCA3*-N103H and pH7WG2-*LHCA4*-N99H vectors were co-transformed into *koLhca3 koLhca4* background, and transformant lines were selected on MS-agar medium supplemented with 50 µg/mL kanamycin and 25 µg/mL hygromycin.

To produce *koLhca3 koLhca4* complemented with WT *(A3WT-A4WT*), Lhca3 *a*603-NH (*A3NH-A4WT*), Lhca4 *a*603-NH (*A3WT-A4NH*), and *a615-HA/HI*, the 5'-UTR, coding sequence and 3'-UTR regions were sequence-optimized for the Golden Braid modular cloning system (Vazquez-Vilar *et al.*, 2017) and obtained as separate synthetic sequences from Genewitz (www.genewiz.com) (Fig. **S28**). The final transcription units encoding for each gene were assembled using type IIS restriction enzymes BsaI and BsmbI (Sarrion-Perdigones *et al.*, 2013). The resulting pDGB3α1-*LHCA3*_H168A-*LHCA4*_H151A and pDGB3α1-*LHCA3*_H168I-*LHCA4*_H151I plant expression vectors were used to complement *koLhca3 koLhca4* plants, and transformant lines were selected on MS-agar medium supplemented with 50 µg/mL kanamycin.

Gel electrophoresis and Immunoblotting

SDS-PAGE analysis was performed using the Tris-Glycine buffer system (Laemmli, 1970) with the modifications described in (Ballottari *et al.*, 2004). For immunotitration, thylakoid samples were loaded for each sample and electroblotted on nitrocellulose membranes. Proteins were detected with primary antibodies (from Agrisera, α-PsaA AS06 172, α-PsbB AS04 038, α-Lhca3 AS01 007, and α-Lhca4AS01 008) and an alkaline phosphatase-conjugated secondary antibody (Sigma-Aldrich A3687). Signal amplitude was quantified using the GelPro 3.2 software.

Phylogenetic analysis

The Timetree in Figure 1a was obtained using TimeTree5 (Kumar *et al.*, 2022).

Fluorescence emission spectra and the fluorescence emission maxima (Fig. **1b**) were recorded at cryogenic temperature. The experimental procedure was as follows: leaf tissue was frozen in liquid nitrogen, ground to a fine powder, and resuspended in measuring buffer (10 mM HEPES pH 7.5, 20% w/v glycerol). Samples were harvested at Padua's Botanical Garden.

Physcomitrium patens (Hedw.) Mitt. was grown as described by (Pinnola *et al.*, 2018). Fluorescence measurements were recorded on functional chloroplasts obtained as described in (Casazza *et al.*, 2001) upon dilution into measuring buffer.

The green algae *Chlamydomonas reinhardtii P.A.Dangeard* and *Chlorella vulgaris Beij.* were cultivated in minimal media, HSM (Sueoka, 1960) and BG-11 (Allen & Stanier, 1968), respectively, until the early exponential phase. They were then diluted into measuring buffer.

For all samples, 500 µL of the suspension was directly used to record the emission spectra.

Protein sequences of *C. reinhardtii* (Cr), *P. patens* (Pp), and *Arabidopsis thaliana (L.) Heynh.)* (At), and *Fittonia albivenis (L.) Ehrh. ex Lindl.* (Fa) Lhca3 and Lhca4 shown in Fig. **1c** were retrieved from the 7ZQC, 7XQP, 9GBI, and 8WGH PDB. For the Lhca3 and Lhca4 protein sequences of *Zea mays L.* (Zm) and *Ananas comosus (L.) Merr* (Ac) were retrieved from Phytozome (PAC:40246382 and PAC:40271081, PAC:33052185 and PAC:33036699, respectively). *Posidonia oceanica (L.) Delile* and

Cymodocea nodosa (Ucria) Asch. Lhca3 and Lhca4 protein sequences were obtained by a *A. thaliana* Lhca3 or Lhca4 tblastn (Altschul *et al.*, 1990) query against the organisms *Posidonia oceanica (L.) Delile* (taxid:55489) and *Cymodocea nodosa (Ucria) Asch.* (taxid:55448) (Database whole genome shotgun sequence). Multiple alignments of the protein sequences were performed with Clustal Omega (Madeira *et al.*, 2022).

Spectral deconvolution of the fluorescence traces in Fig. **1b** was performed by fitting four Gaussian curves using an in-house Python script. The initial guesses for the four central peaks were 680 nm, 690 nm, 732 nm, and 750 nm. The fraction of the area under the curve at wavelengths ≥720 nm was obtained by dividing the sum of the areas of the Gaussians above 720 nm by the sum of the areas of the four fitted Gaussians.

Structure refinement for QM/MM calculations

All calculations for the WT and *a*603-NH mutant species were performed on the Cryo-EM structures presented in this work. Water molecules coordinating the Chlorophyll pigments (Chls) were added to the structures by homology with a previously published structure (see below). Indeed, while waters were not resolved in the density map at this resolution, their presence deeply affects the excitation energies of nearby Chls and therefore must be considered. Six water molecules were added: five of them were taken from the WT structure of *Pisum Sativum* (Wang *et al.*, 2021) coordinating Chls 304, 306, 307, 308 (two H-bonded water molecules); one more water molecule to coordinate the Mg center of Chl 311, which was lacking a coordination residue.

Some of the mobile phytyl tails of the Chls, which were not resolved in the structure, were rebuilt with the *tleap* tool of the Amber (Case *et al.*, 2020) suite and clashes with the rest of the structure were minimized by iteratively rotating the dihedral angles of the added segments through a Monte Carlo scheme. All protein residues were kept in their standard protonation, except for Mg-coordinating HIS, which were forced to be delta-protonated to ensure the metal binding. Moreover, residue Glu 146 was protonated to allow the formation of an H-bond with the carbonyl group of Chl 307, as in (Sláma *et al.*, 2023). The added segments, water molecules, and H atoms were then relaxed by MM minimization with constraints on the rest of the structure. The Amber ff14SB force-field was employed for the protein, and the TIP3P model to describe water molecules. The parameters for the cofactors Chls, 1,2-distearoyl-monogalactosyl-diglyceride (LMG), beta-carotene (BCR), lutein (LUT), and violaxantin (XAT) were taken from previous work in the group (Prandi *et al.*, 2016).

A structure refinement protocol was then applied to the prepared system to achieve a proper description of the pigment geometries and of the protein pockets. The refinement protocol comprises three steps:

(1) restrained relaxation of all heavy atoms; (2) multiple cycles of simulated annealing runs; (3) QM/MM optimizations (Chung *et al.*, 2015) of the Chls.

During Step 1, all the structure was relaxed with positional restraints of 10 kcal/mol-A^2 and subsequently, in a second step, of 4 kcal/mol-A^2 on the protein backbone atoms and on heavy atoms of XAT, LMG, LUT (only the two extrema of the conjugated chain), and finally on the internal ring connecting N atoms and on Mg for Chls. Protein residues coordinating the Chls were left free to move to adjust the coordination geometry. To compensate for force-field inaccuracies in describing the Mg-binding residue distances, fictitious bonds between the Mg atoms and their binding residues were added, whose equilibrium distances were assigned based on average binding distances obtained on QM/MM-optimized structures.

Step 2 consisted of 5 cycles of simulated annealing: a T ramp of 0 K - 300 K - 0 K and the Berendsen thermostat were employed. The final structure of each run was used as a starting point for the following one; the structure of the last run was finally MM-minimized. Positional restraints of 10 kcal/mol-A^2 analogous to the ones of the previous step were used during simulated annealing to keep the overall structure close to the crystal one, while allowing the side chains to rearrange towards a more favorable conformation. In the case of the mutant structure, smaller restraints were imposed to the *a*603 ring to allow it to rearrange to the more hindered pocket.

Within Step 3, the Chls' internal geometry was optimized at QM/MM level using an ONIOM scheme. Each Chl molecule was independently optimized; the high-level layer of the ONIOM scheme comprised the Chl ring and the binding residue (protein sidechains or water molecules) and was allowed to move. The low-level layer included the protein environment within 25 A from the Chl molecule and was frozen during optimization. The optimizations were run in Gaussian 16 at B3LYP/6-31G(d) level of theory (Frisch *et al.*, 2016).

The *a*603-*a*609 Chl dimer was further refined together in order to achieve an accurate description of the intermolecular conformation. Indeed, it is known that CT excitations are extremely sensitive to the relative position and orientation of the pigments. A new ONIOM optimization was run, including the two Chl rings and three protein residues of the pocket (His 99, Glu 154 and Arg 157) as the moving high-level layer, and the environment within 25 A as the low-level layer (kept frozen as before).

Before calculation, the final refined models were compared with the experimental Cryo-EM structures. After relaxation/minimization, only minimal changes were observed both in the main chain (Ca-RMSD of 0.12 and 0.13 for WT and mutant N98H, respectively) and in the position of the chlorophyll rings and carotenoids (Fig. **S29**).

Excited-state calculations

We computed all elements (site energies and couplings) of the exciton Hamiltonian of Lhca4 on the structure refined as detailed above. The site energies of the individual chlorophylls were computed at the TD-DFT M062X/6-31G(d) level of theory. This choice is based on previous works on LHCs (Sláma *et al.*, 2020; Saraceno *et al.*, 2023). The environmental effects were included through a polarizable QM/MM methodology (QM/MMPol): the QM part is composed of the chlorophyll ring (a H atom is added to saturate the valence after cutting the phytyl tail), while the rest of the protein is treated at the polarizable MM level. The charges and polarizabilities for the MM part were the same as in (Sláma et al., 2023). The Chl-Chl exciton couplings were calculated using the TrEsp (transition charges from electrostatic potentials) approach (Madjet *et al.*, 2006), wherein the transition charges required to compute the couplings are obtained from a fit of the electrostatic potential generated by the transition density. All QM/MMPol calculations were run using a locally modified version of Gaussian 16.

Next, to include the charge-transfer (CT) states, we built an extended Hamiltonian with CT energies and couplings. The CT states were computed for two supramolecules: (i) the *a*603-*a*609 dimer and (ii) the trimer, *a*603-*a*609-vio. For both cases, the excited states were computed on the refined structure using the Tamm–Dancoff approximation (TDA) formulation of TD-DFT at ωB97XD/6-31G(d) level of theory within the QM/MMPol scheme. ωB97XD was chosen because a good description of CT states requires the use of long-range corrected functionals. For case (i), the QM part consisted of the chlorophyll dimer, *a*603 and *a*609, while for case (ii), the QM part included Vio along with *a*603-*a*609 dimer. To obtain the CT energies and the corresponding locally excited (LE) state-CT couplings from the excited state calculations, the multistate-FED-FCD diabatization scheme (Nottoli *et al.*, 2018) was employed on the first 14 excited states of the dimer. The multi-FED-FCD scheme combines the Fragment Charge Difference (FCD) and Fragment Excitation Difference (FED) methods to separate LE/CT subspaces for multiple states and extract electronic couplings. The two lowest CT states, $a603^{+}a609^{-}$ and $a603^{-}a609^{+}$, obtained from this diabatization scheme, were added to the exciton Hamiltonian to build the extended Hamiltonian. The site energies and couplings in the exciton Hamiltonian corresponding to *a*603 and *a*609 are replaced by the LE energies and couplings from diabatization. To correct for the difference in level of theory between the site and CT calculations while building the extended Hamiltonian, we calculate the shift in the average of the LE state energies (*a*603* and *a*609*) from the diabatization calculation with respect to the average of the site energies and add this shift to the CT and LE energies in the extended Hamiltonian.

Simulation of absorption spectra

The spectrum of the excitonic aggregate was simulated in the disordered exciton model by summing contributions of absorption to individual exciton states. Static disorder was included as Gaussian

random shifts of the pigments' site energies and CT energies (600 realizations, with a standard deviation of 100 cm^{-1} for LE states and 1000 cm^{-1} for CT states). The homogeneous absorption lineshape of each exciton is obtained using the spectral density formalism and the second-order cumulant expansion in the so-called complex Redfield approximation (Gelzinis *et al.*, 2015). An experimentally-derived spectral density was used to describe coupling to vibrations localized on the pigment and on the protein environment (Novoderezhkin *et al.*, 2004).

All spectra were simulated using the in-house pyQME package (https://github.com/Molecolab-Pisa/pyQME).

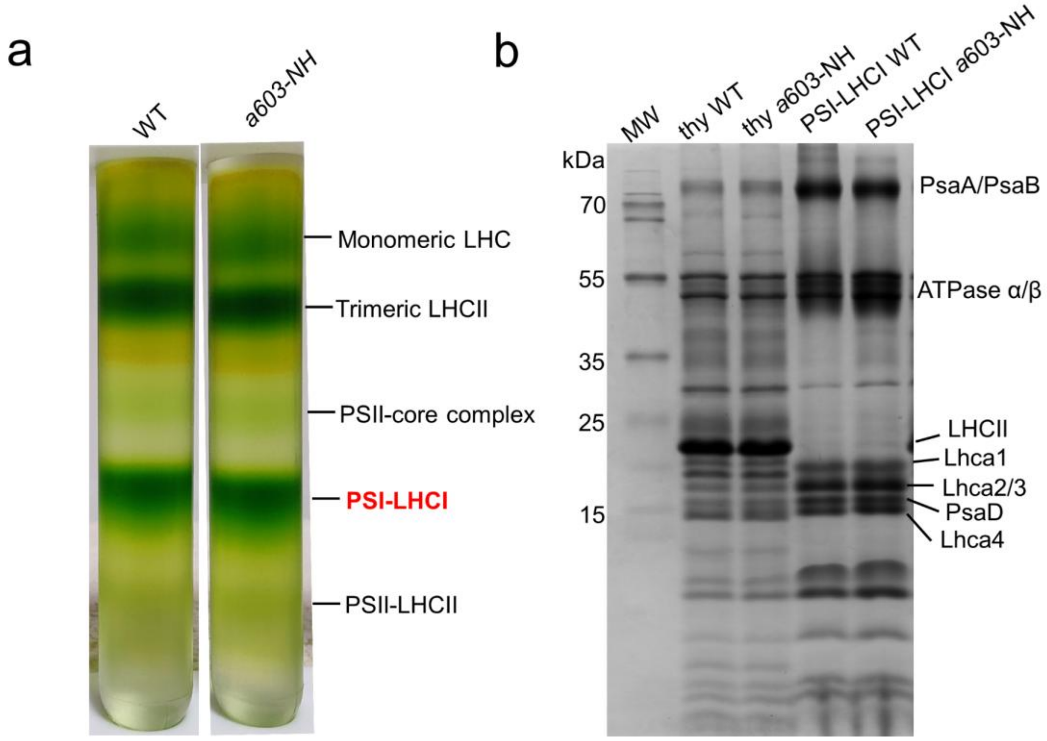

Figure S1: Sample preparation and characterization. (a) Sucrose density gradient fractionation of solubilized thylakoid membranes from *Arabidopsis thaliana* WT and *a*603-NH mutant strains. The PSI-LHCI bands harvested for Cryo-EM and further characterization are labeled in red. (b) SDS-PAGE analysis of thylakoid membranes (thy) and PSI-LHCI supercomplexes of WT and *a*603-NH mutant.

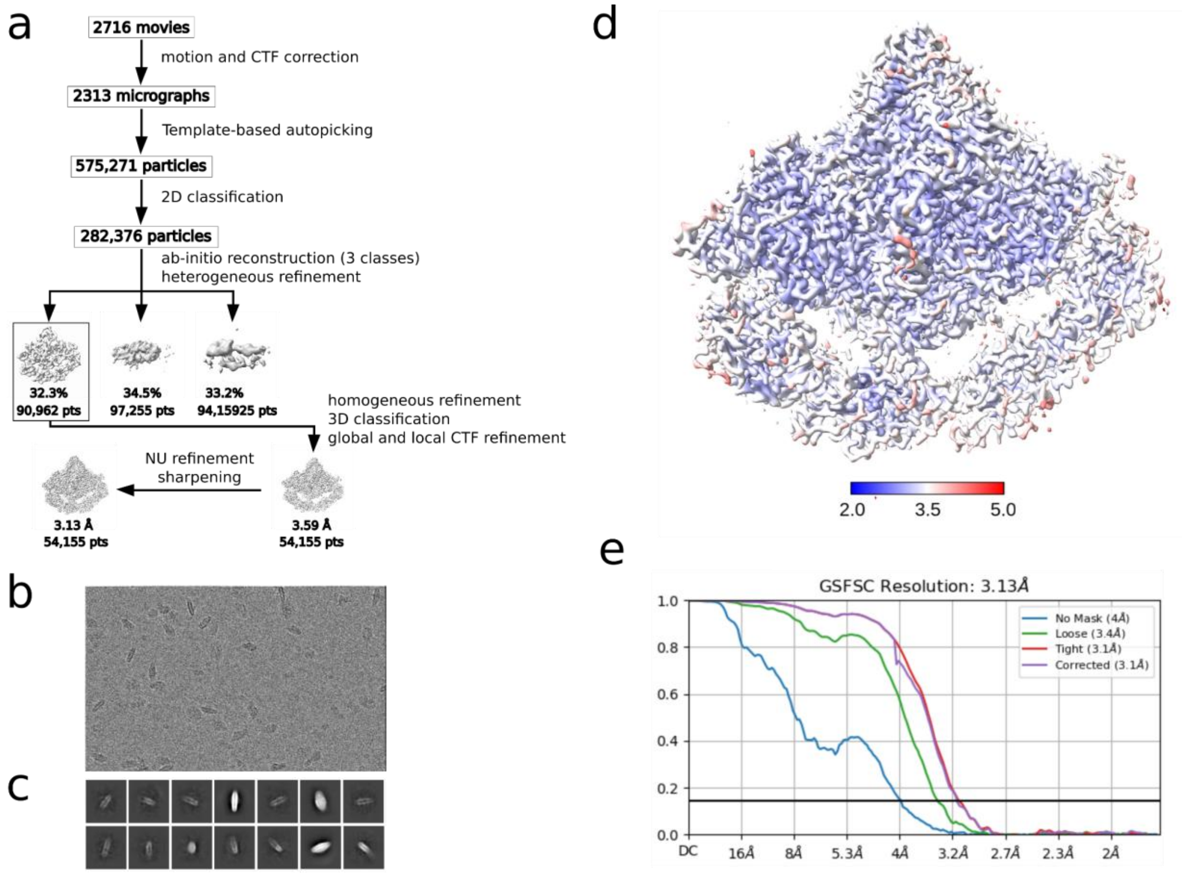

Figure S2: Cryo-EM workflow, images and map quality for AtPSI-LHCI WT. (a) Cryo-EM data processing workflow for AtPSI-LHCI WT. Representative Cryo-EM micrograph (b) and 2D class averages (c) of the supercomplex. (d) Estimation of the local resolution of the final map. e) Gold standard Fourier shell correlation (GSFSC) curve of the final reconstruction.

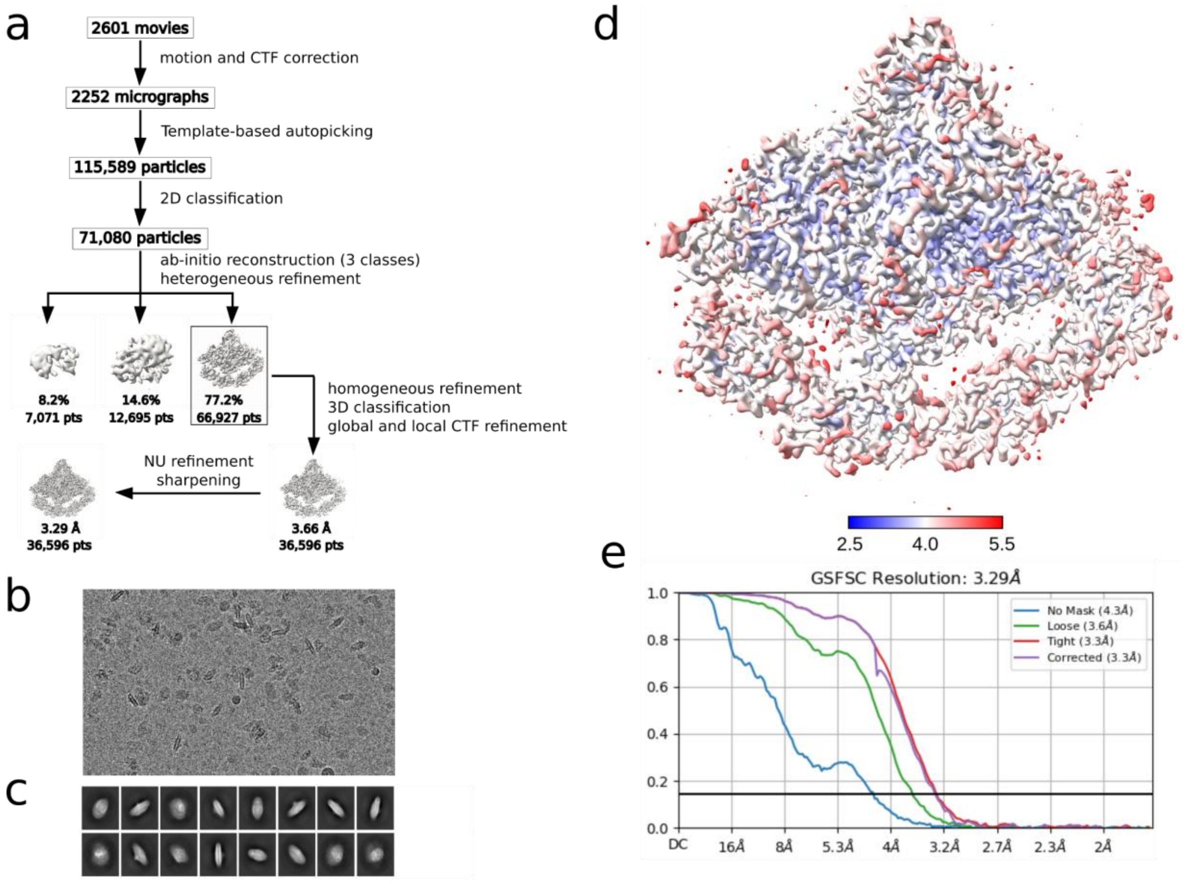

Figure S3: Cryo-EM workflow, images and map quality for AtPSI-LHCI *a*603-NH. (a) Cryo-EM data processing workflow for AtPSI-LHCI *a*603-NH. Representative Cryo-EM micrograph (b) and 2D class averages (c) of the supercomplex. (d) Estimation of the local resolution of the final map. e) Gold standard Fourier shell correlation (GSFSC) curve of the final reconstruction. Cryo-EM workflow for AtPSI-LHCI *a*603-NH.

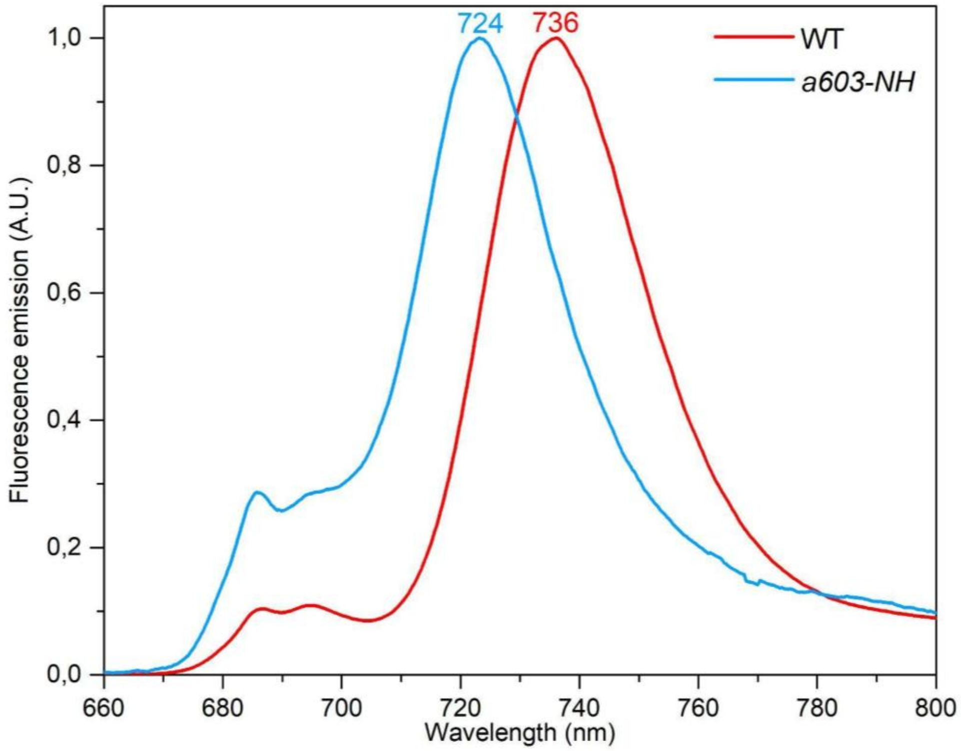

Figure S4: Emission spectra of leaves of *A. thaliana* WT and *a*603-NH mutant. Fluorescence emission spectra were measured on intact leaves from WT, and *a*603-NH mutant (a603-NH) and normalized to the λmax.

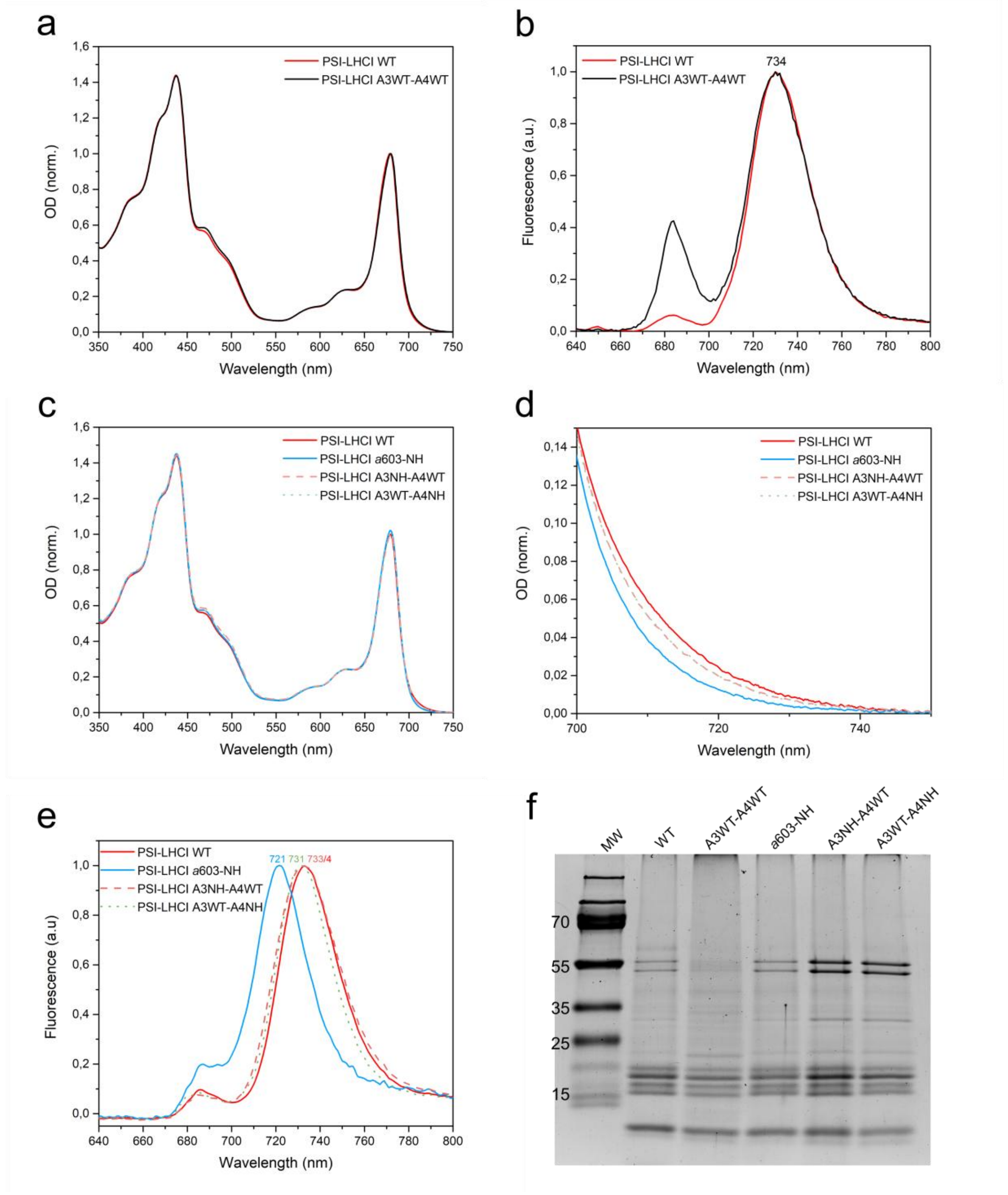

Figure S5: Characterization of PSI-LHCI purified from WT, A3WT-A4WT, A3NH-A4WT and A3WT-A4NH mutant lines. (a) RT absorption spectra of PSI-LHCI WT and A3WT-A4WT. (b) 77K emission spectra of PSI-LHCI WT and A3WT-A4WT. (c) RT absorption spectra of PSI-LHCI WT, A3NH-A4WT and A3WT-A4NH (d) zoom in the λ≥ 700 nm region of RT absorption spectra of PSI-LHCI WT, A3NH-A4WT and A3WT-A4NH. (e) 77K emission spectra of PSI-LHCI WT, A3NH-A4WT and A3WT-A4NH (f) SDS-PAGE analysis of PSI-LHCI purified from WT, A3WT-A4WT, A3NH-A4WT, and A3WT-A4NH mutant lines.

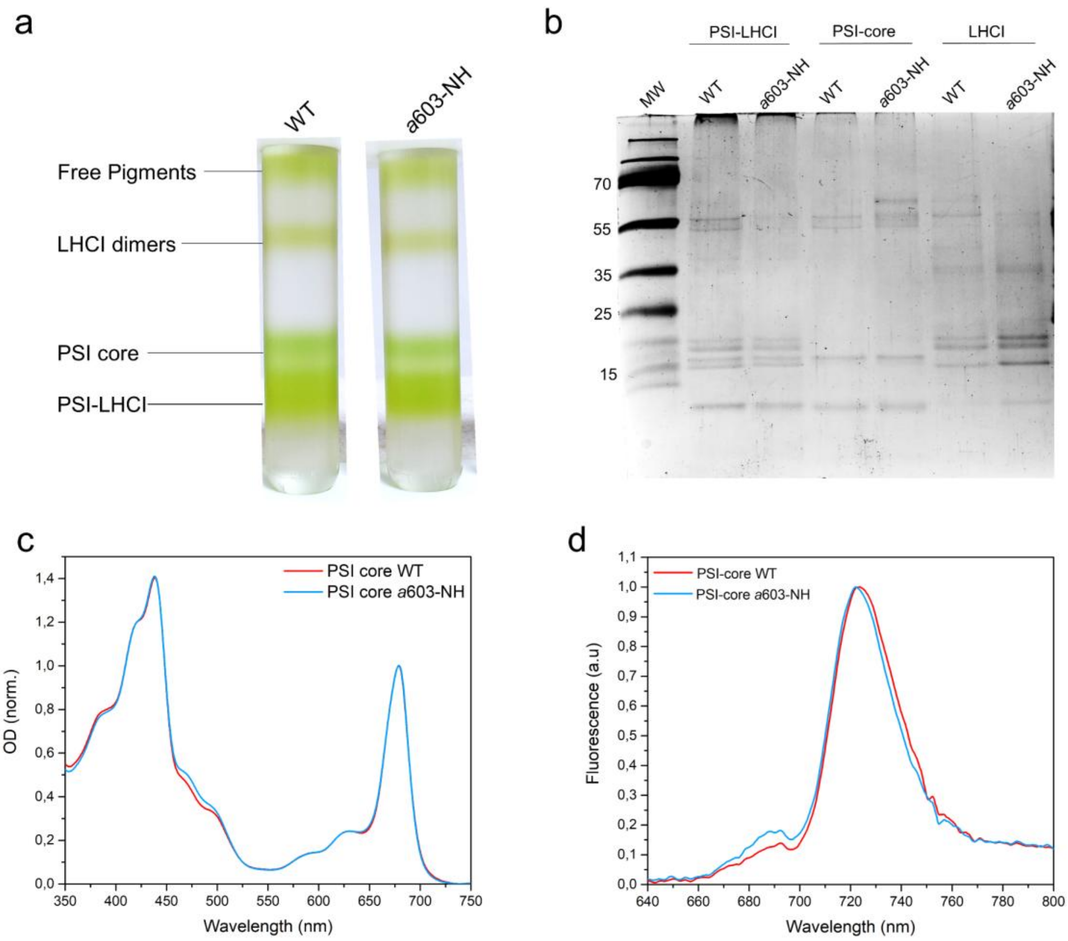

Figure S6: Characterization of solubilized PSI-LHCI from WT and *a*603-NH. (a) Sucrose gradient fractionation of solubilized PSI-LHCI from WT and *a*603-NH plants (b) SDS-PAGE analysis of sucrose bands fractions (c) Room temperature (RT) absorption of the PSI core from *A. thaliana* WT and *a*603-NH. (d) 77K fluorescence emission spectra of the PSI core from *A. thaliana* WT and *a*603-NH.

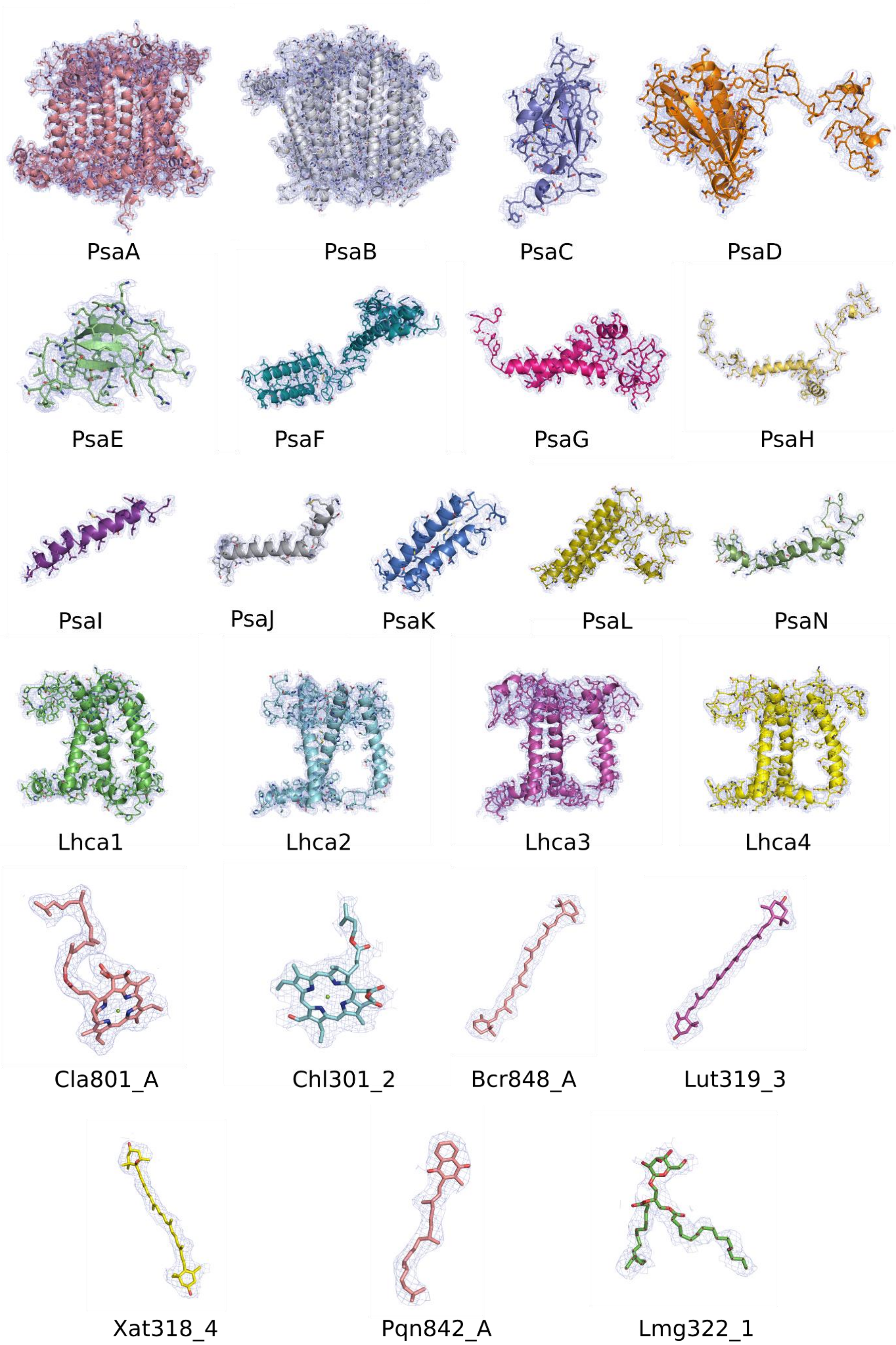

Figure S7: Atomic models of PSI-LHCI subunits and selected ligands superimposed on Cryo-EM maps.

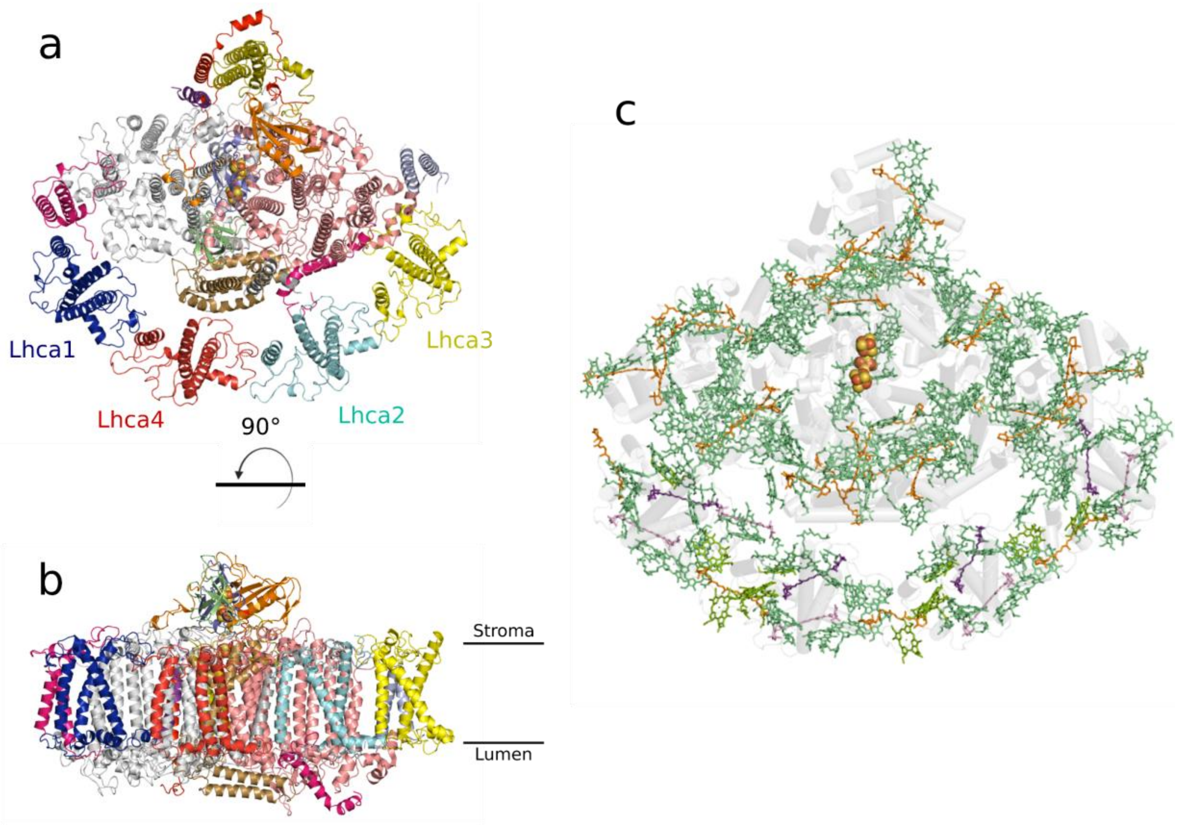

Figure S8: Overall architecture of the PSI-LHCI WT supercomplex. (a-b) Top and side view of the PSI-LHCI WT supercomplex. Different subunits are colored with different colors. (c) Pigment organization in the PSI-LHCI WT of *A.thaliana* (PDB 9GBI): Chl *a* (green), Chl *b* (light green), Violaxanthin (purple), β-carotenes (orange), and Lutein (pink).

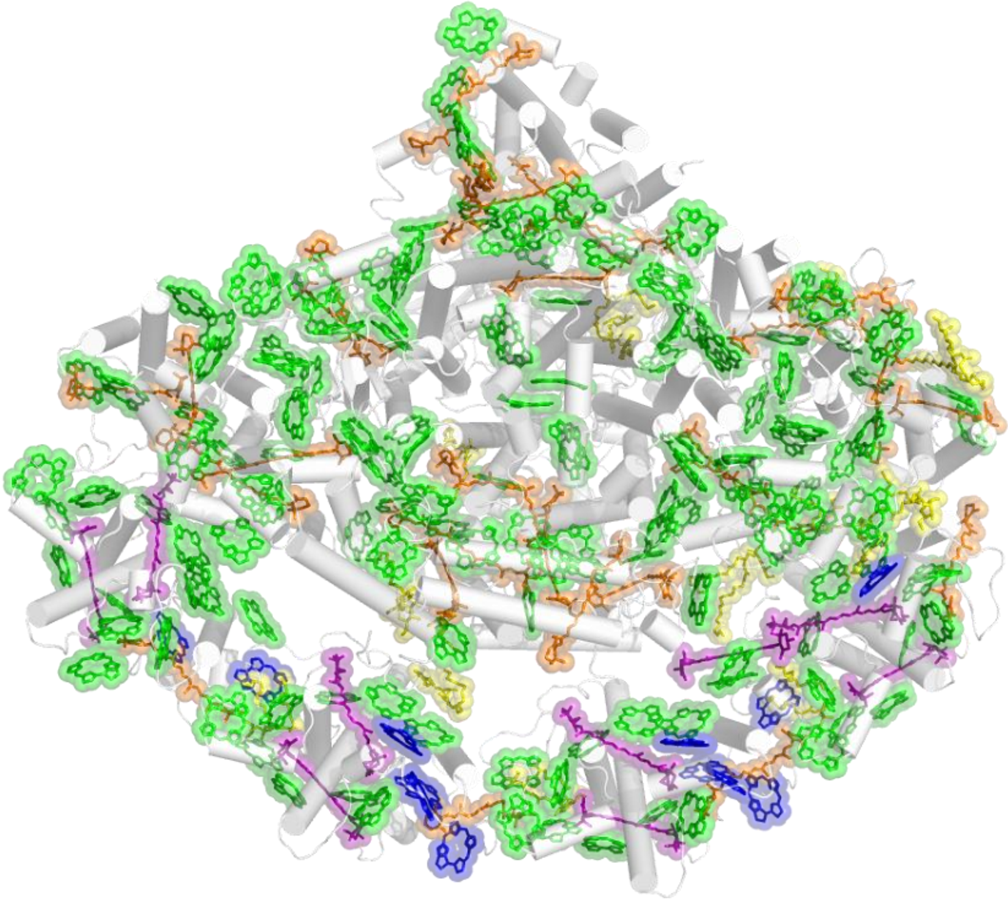

Figure S9: **Positions of ligands in the PSI-LHCI WT of *A.thaliana* (PDB 9GBI).** Color codes Chl *a* (green), Chl *b* (blue), Xanthophylls (purple), β-carotenes (orange), and lipids (yellow).

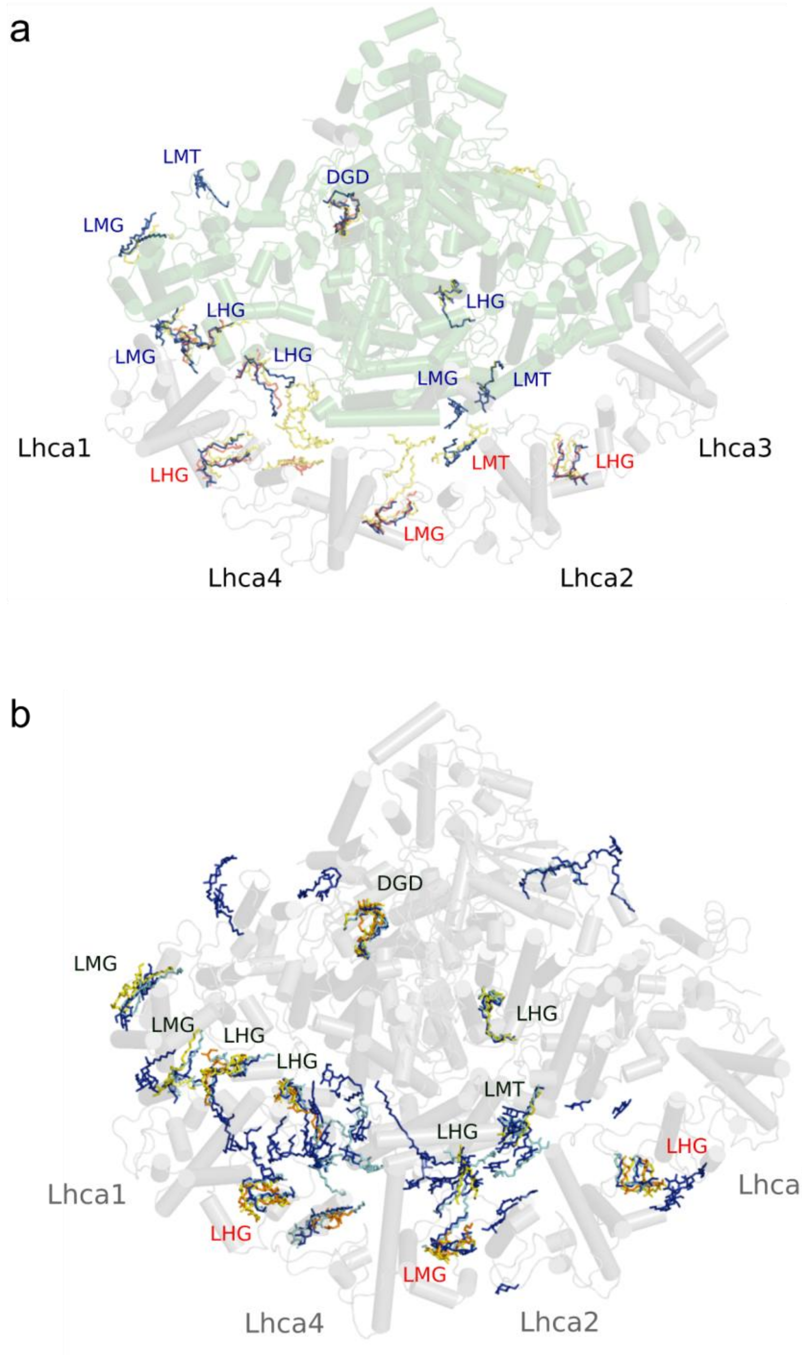

Figure S10: Lipid arrangement in PSI-LHCI supercomplexes. (a) Lipids found in the structures of AtPSI-WT (PDB 9GBI, blue), PDB 8JZB (red), and PDB 7DKZ (yellow). DGD: digalactosyl-diacyl glycerol (DGDG), LMG: 1,2-distearoyl-monogalactosyl-digliceride, LHG: 11,2-dipalmitoyl-phosphatidyl-glycerol, LMT: dodecyl-β-D-maltoside. (b) Lipids found in the structures of AtPSI-603-NH (PDB 9GC2, yellow), PDB 8JZB (orange), PDB 7DKZ (cyan), and PDB 5L8R (blue). DGD: digalactosyl-diacyl glycerol (DGDG), LMG: 1,2-distearoyl-monogalactosyl-digliceride, LHG: 11,2-dipalmitoyl-phosphatidyl-glycerol, LMT: dodecyl-β-D-maltoside.

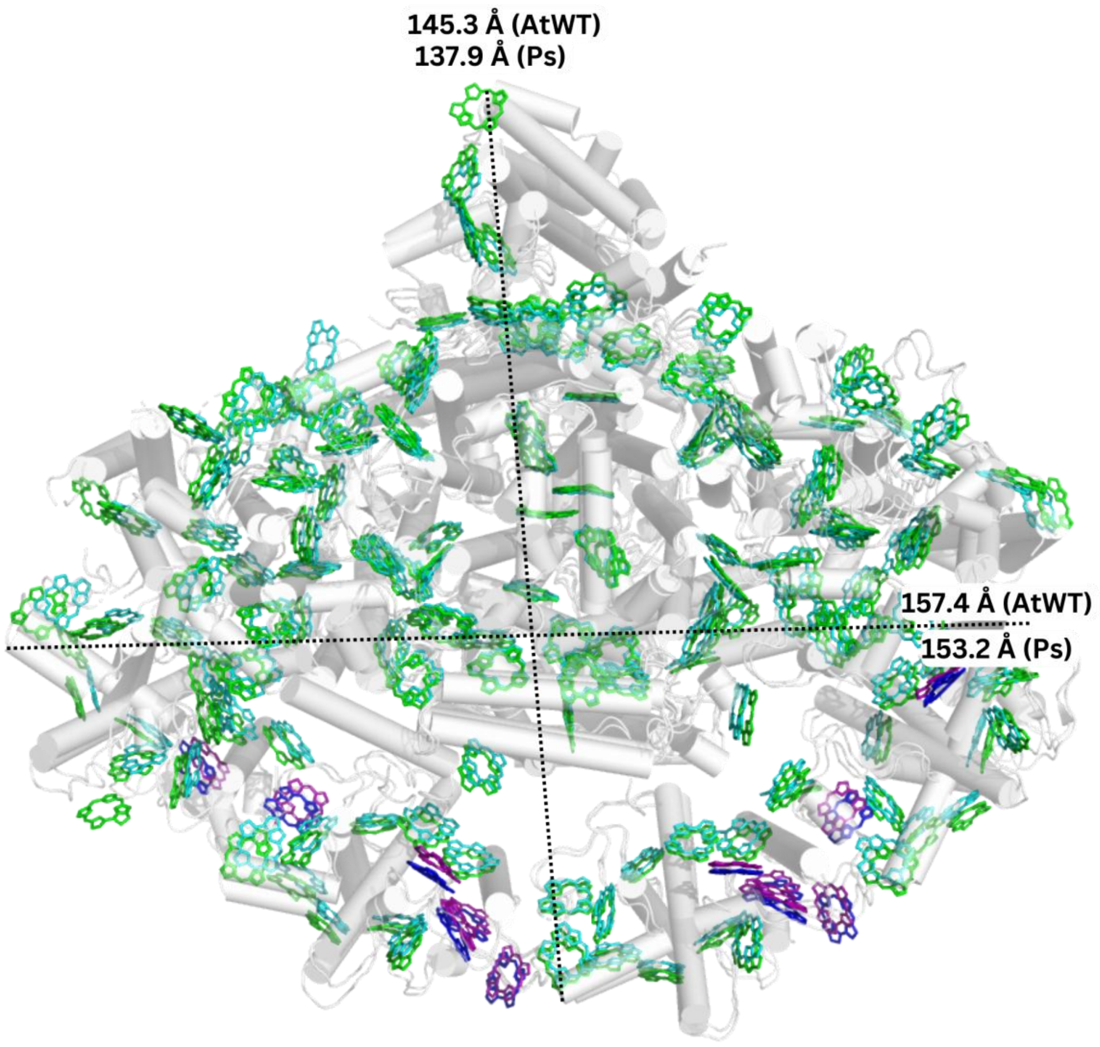

Figure S11: **Superposition of the PSI WT from *A. thaliana* (PDB 9GBI, white) and from *P. sativum* (PDB 7DKZ).** chl *a* from *A. thaliana* and *P. sativum* are colored green and cyan, respectively. chl *b* from *A. thaliana* and *P. sativum* are colored in blue and purple, respectively. The diameter lengths are reported in Å.

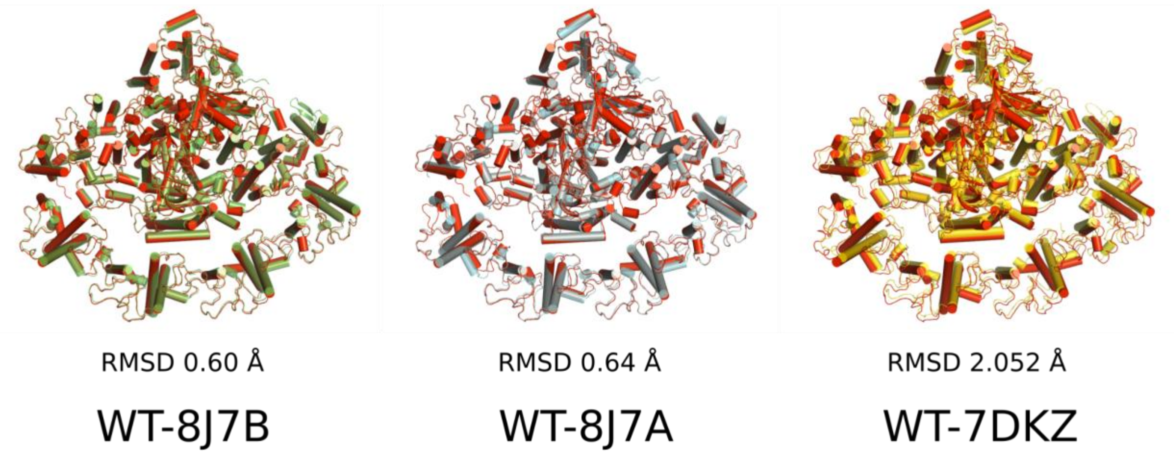

Figure S12: Global superposition RMSD of the AtPSI-WT (PDB 9GBI) structure with PSI-WT structures from PDB 8J7B, 8JZA (both from Cryo-EM data), and 7DKZ (from crystallographic data).

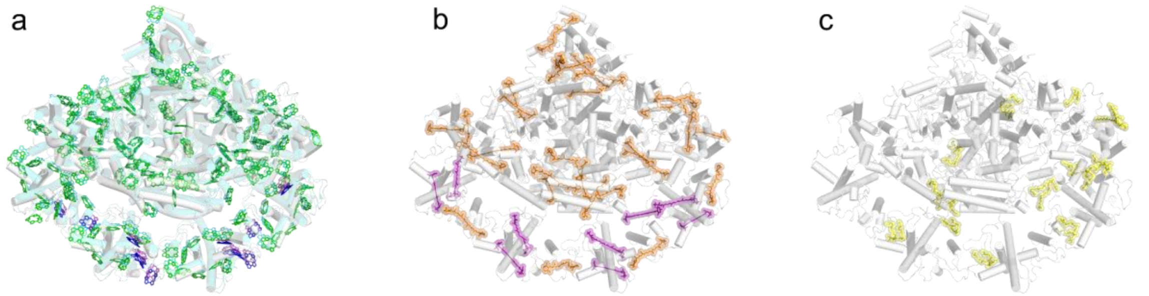

Figure S13: **Chlorophyll, xanthophyll and lipid arrangement in PSI-LHCI WT.** (a) Positions of Chl *a* (green) and Chl *b* (blue) in the PSI-LHCI WT of *A.thaliana*. (b) Positions of Xanthophylls (purple) and β-carotenes (orange) in the PSI-LHCI WT of *A. thaliana*. (c) Positions of lipids (yellow) in the PSI-LHCI WT of *A. thaliana* (PDB 9GBI).

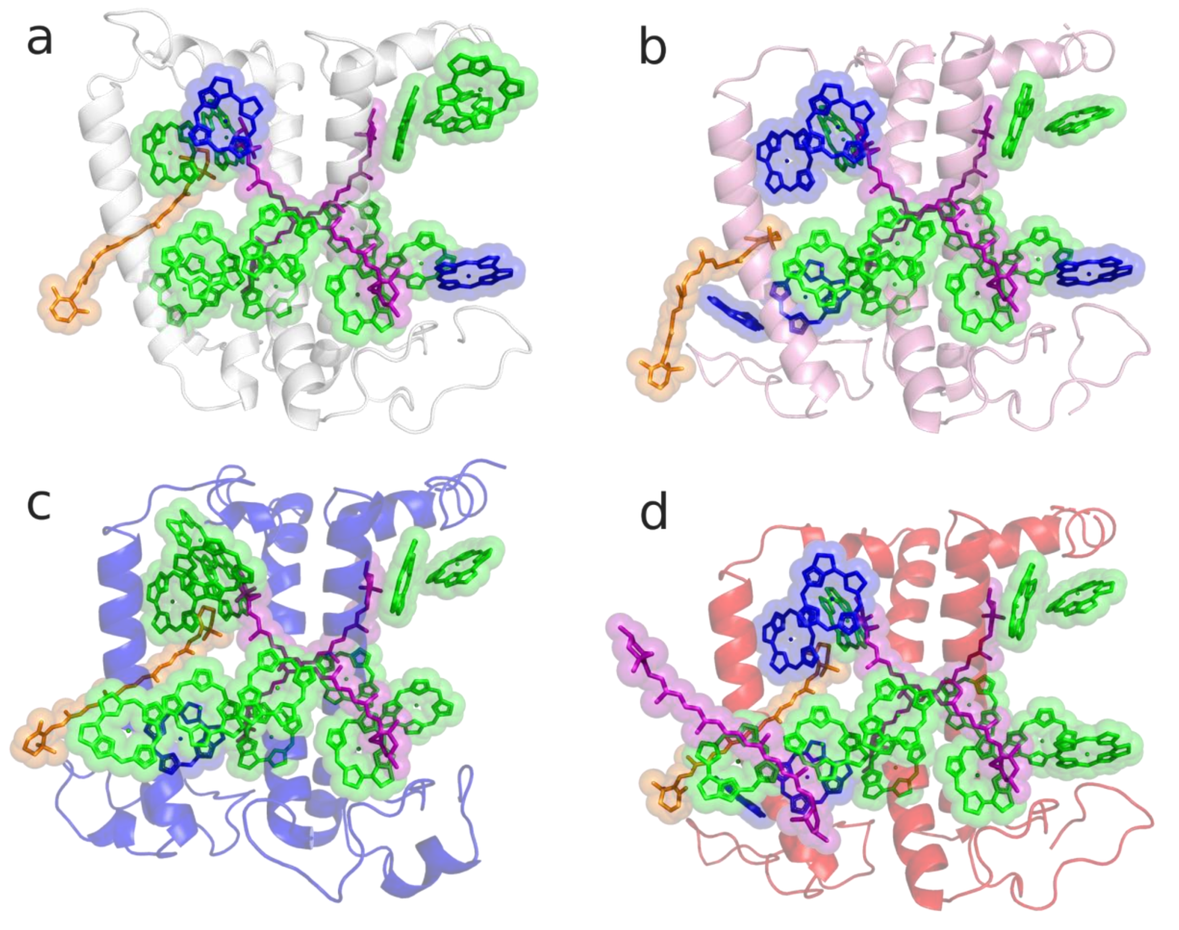

Figure S14: Pigment content of the LHCI antenna subunits of *A. thaliana* WT (PDB 9GBI). (a) Lhca1, (b) Lhca2, (c) Lhca3, (d) Lhca4. Chl *a*, Chl *b*, xanthophylls, and β-carotenes are colored green, blue, purple, and orange, respectively. The type and position of pigments visible in the LHCI subunits of the *a*603-NH structure are virtually identical to those of the WT structure (except for the position of Chl *a*603 in Lhca3 and Lhca4) and therefore are not shown.

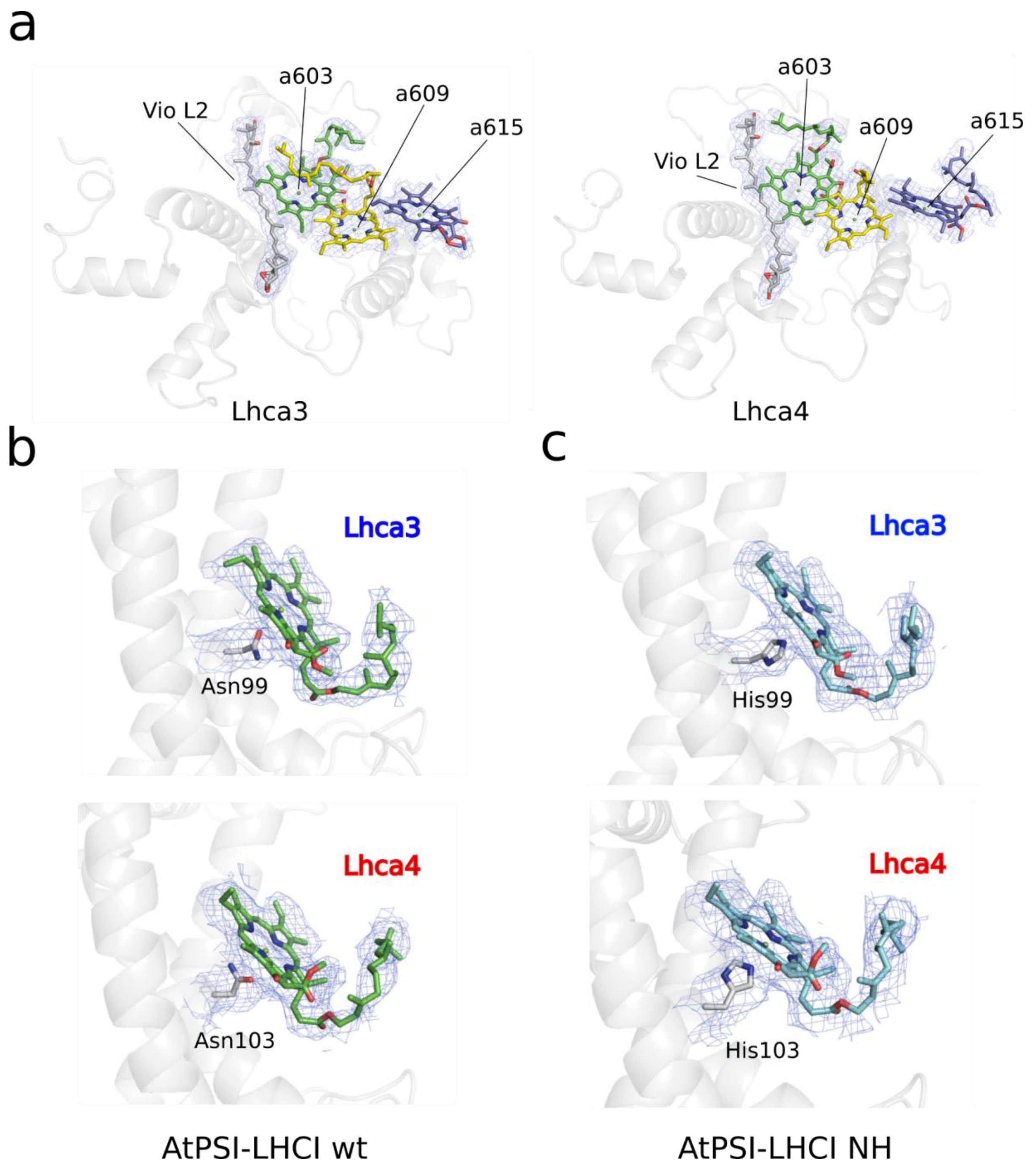

Figure S15: Atomic models of the "red cluster" pigments. (a) Red cluster pigments in Lhca3 (left) and Lhca4 (right) are shown surrounded by Cryo-EM maps. (b-c) The position of Chl *a*603 and its axial ligand (stick models) in Lhca3 and Lhca4 (WT and *a*603-NH) surrounded by Cryo-EM maps.

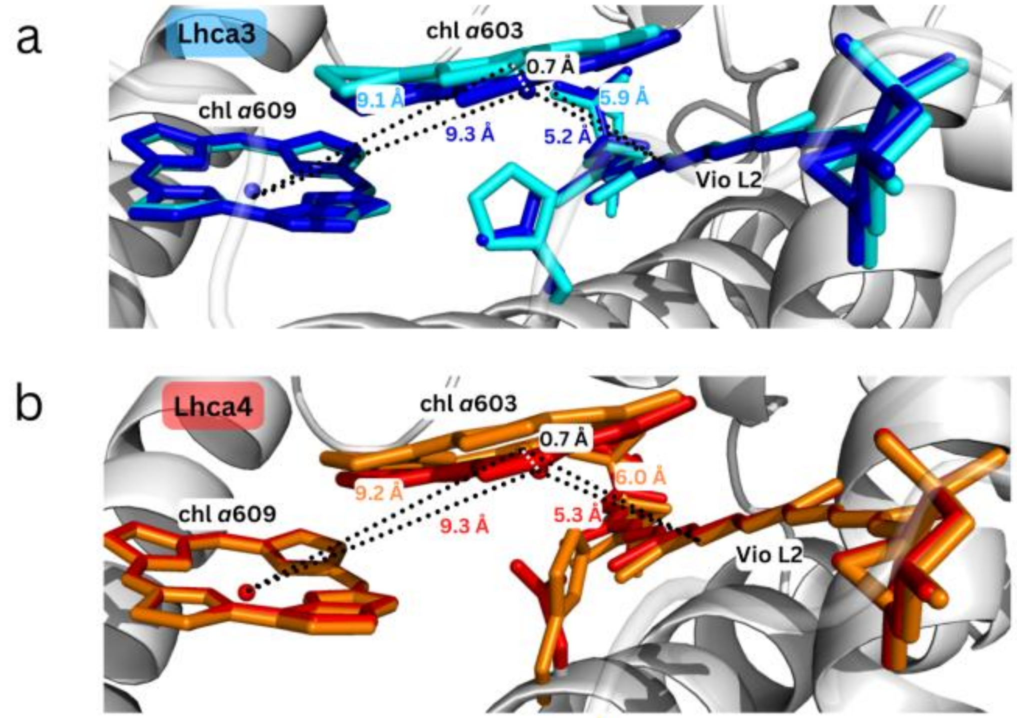

Figure S16: Distances between Chl *a*603, Chl *a*609, and Violaxanthin L2 in Lhca3 (a) and Lhca4 (b) subunits of *A. thaliana*. WT (PDB 9GBI) is colored blue and red, and *a*603-NH (PDB 9GC2) is colored cyan and orange. The superposition was obtained by aligning the Cα position of Lhca3 (a) or Lhca4 (b) *a*603-NH with the corresponding chain of WT.

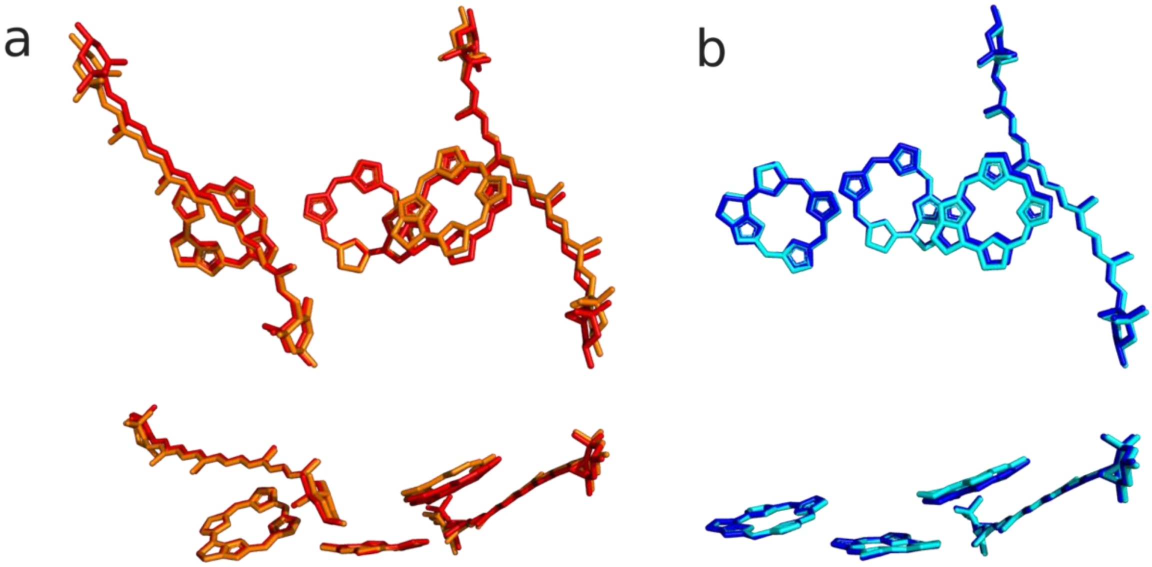

Figure S17: Superposition of selected Chls and xanthophylls of the Lhca3/Lhca4 WT and *a*603-NH. Top and side view of superimposed structures of Chl *a*603, Chl *a*609, Chl *a*615, Violaxanthin L2 and Lutein from (a) Lhca4 WT and *a*603-NH (red and orange, respectively) and (b) Lhca3 WT and *a*603-NH (blue and cyan, respectively). Pigments were superimposed on Chl *a*609.

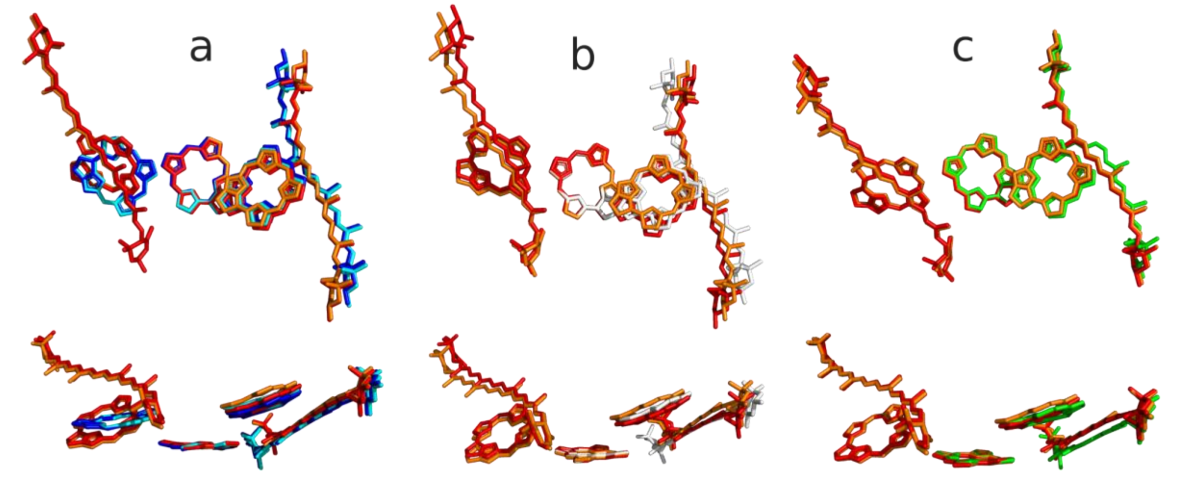

Figure S18: Superposition of selected Chls and xanthophylls of the Lhca1-4 WT and *a*603-NH. Top and side view of superimposed structures of Chl *a*603, Chl *a*609, Chl *a*615, Violaxanthin L2 and Lutein from (a) Lhca4 WT and *a*603-NH (red and orange, respectively) with Lhca3 WT and *a*603-NH (blue and cyan, respectively), (b) Lhca4 WT and *a*603-NH (red and orange, respectively) with Lhca1 WT (white), and c) Lhca4 WT and *a*603-NH (red and orange, respectively) with Lhca2 WT (green). Pigments were superimposed on Chl *a*609.

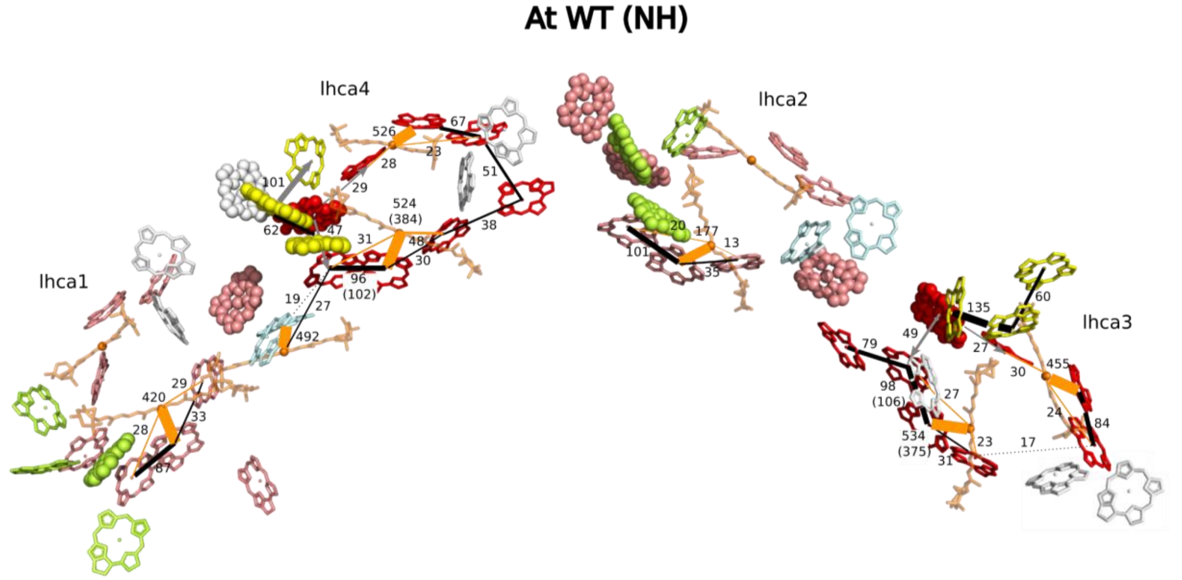

Figure S19: Excitonic coupling absolute values between pigments in LHCI WT and *a*603-NH (values in parenthesis). All values are in cm^{-1}. Couplings involving xanthophylls and carotenoids are colored orange. The thickness of the line is proportional to the value of the coupling. Couplings with absolute values higher or lower than 20 cm^{-1} are indicated with a solid or dotted line, respectively. Chlorophylls *b* are represented as spheres. Chlorophylls are grouped and colored by clusters wherever the couplings between them are lower than 20 cm^{-1}.

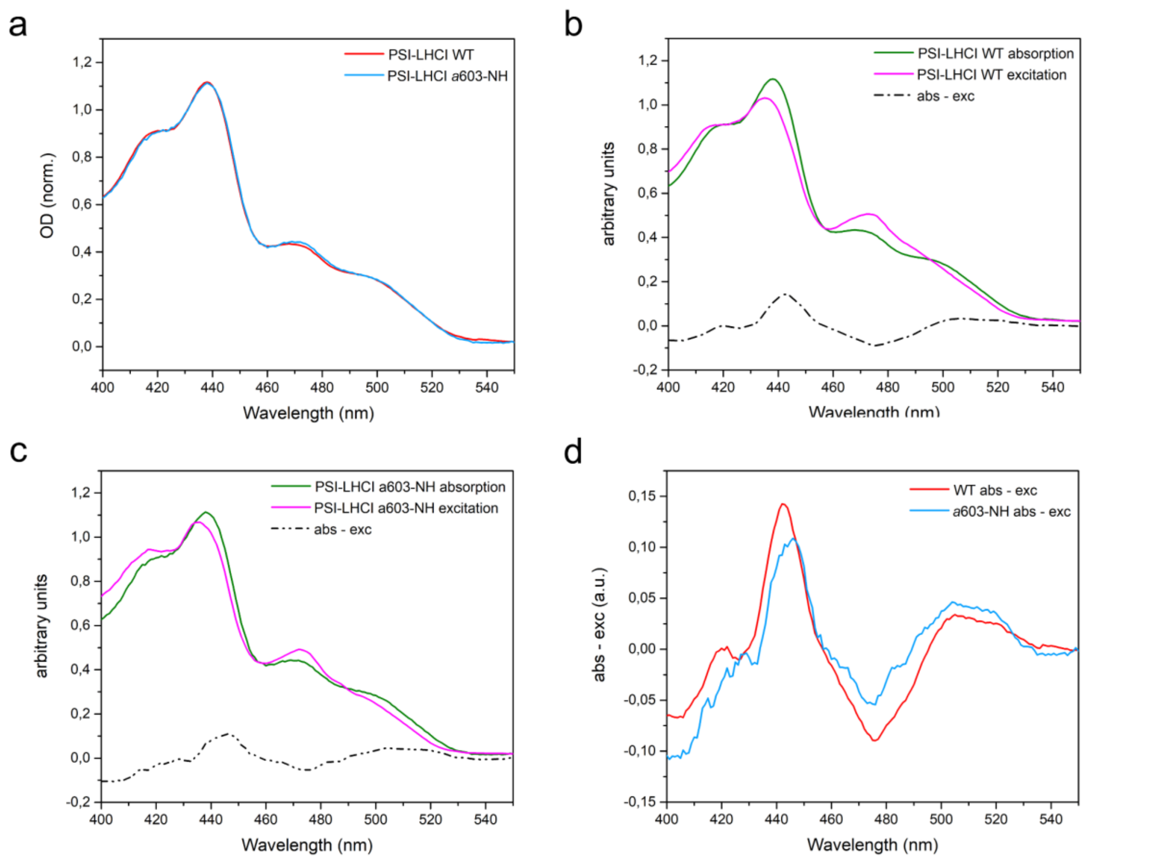

Figure S20: Difference spectra (absorption - excitation spectra, measured at 77K in the 400-550 nm region) of PSI-LHCI from *A. thaliana* WT and *a*603-NH. (a) 77K absorption spectra (400-550 nm) of WT and *a*603-NH PSI-LHCI supercomplex. (b) superimposition of absorption (green) and excitation (magenta) spectra of WT PSI-LHCI (c) superimposition of absorption (green) and excitation (magenta) spectra of *a*603-NH PSI-LHCI (d) 77K difference spectra (abs - exc) of WT and *a*603-NH mutant PSI-LHCI. All the spectra are normalized to the integrated area under the curve between 400 and 550 nm. Spectral traces were calculated as the average of 3 replicates.

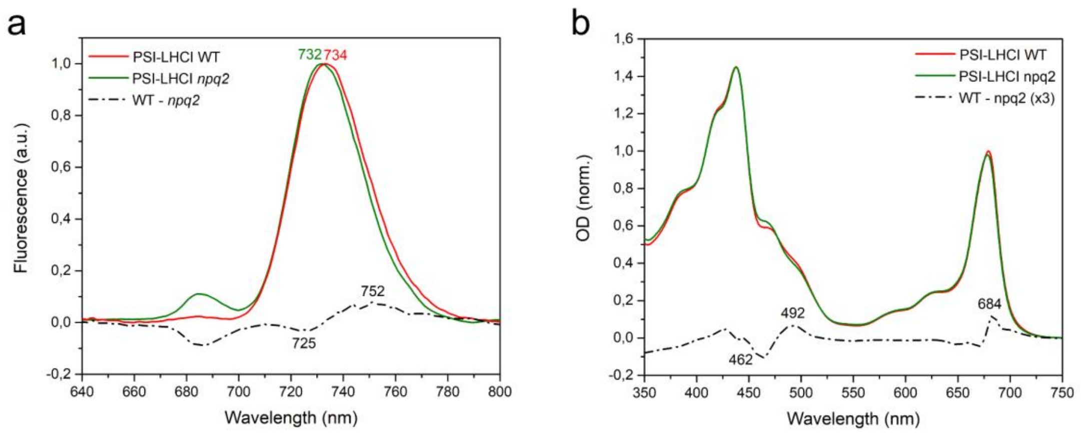

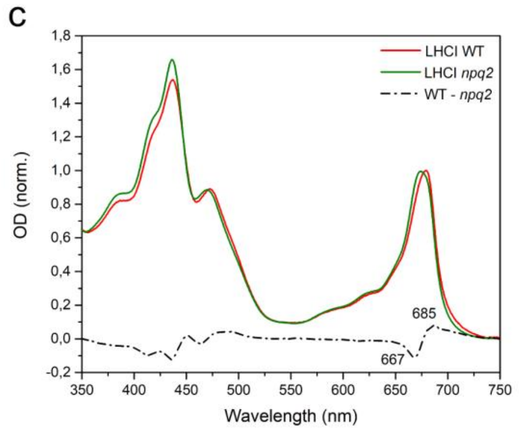

d

Pigment Composition	LHCI WT	*LHCI a*603-NH	*LHCI npq2*
Chl *a/b*	3,5 ± 0,1	3,4 ± 0,5	3,5 ± 0,3
Chl/Car	4,7 ± 0,1	4,4 ± 0,1	5,0 ± 0,3
Vio	3,9 ± 0,1	4,5 ± 0,5	-
Zea	-	-	6,1 ± 0,2
Lut	10,9 ± 0,2	11,1 ± 1,7	6,8 ± 0,2
β-Car	6,6 ± 0,6	7,1 ± 1,8	7,0 ± 1,3

Figure S21: Spectral analysis and pigment composition of PSI-LHCI from WT and *npq2*. (a) Fluorescence emission spectra of PSI-LHCI from *A. thaliana* WT and *npq2* strains, normalized on the reddest peak. Samples were excited at 440 nm, and emission spectra were recorded from 640 to 800 nm: (b, c) RT absorption spectra of PSI-LHCI and LHCI from *A. thaliana* WT and *npq2* lines, normalized on the absorption area between 550 and 750 nm. (b) The difference spectra are shown as black lines in the corresponding plots. The values of the absorption difference spectrum have been magnified by a factor of 3 (panel b) for better visualization. Key wavelengths corresponding to absorption/emission peaks are indicated in nm above the respective peaks. (d) Pigment composition of LHCI complexes purified from WT, *a*603-NH and *npq2*. Quantification was carried out by fitting acetonic spectra and HPLC separation (n=3 independent preparations). Car content was normalized on 100 Chls *a* + *b*. Values represent mean ± standard deviation.

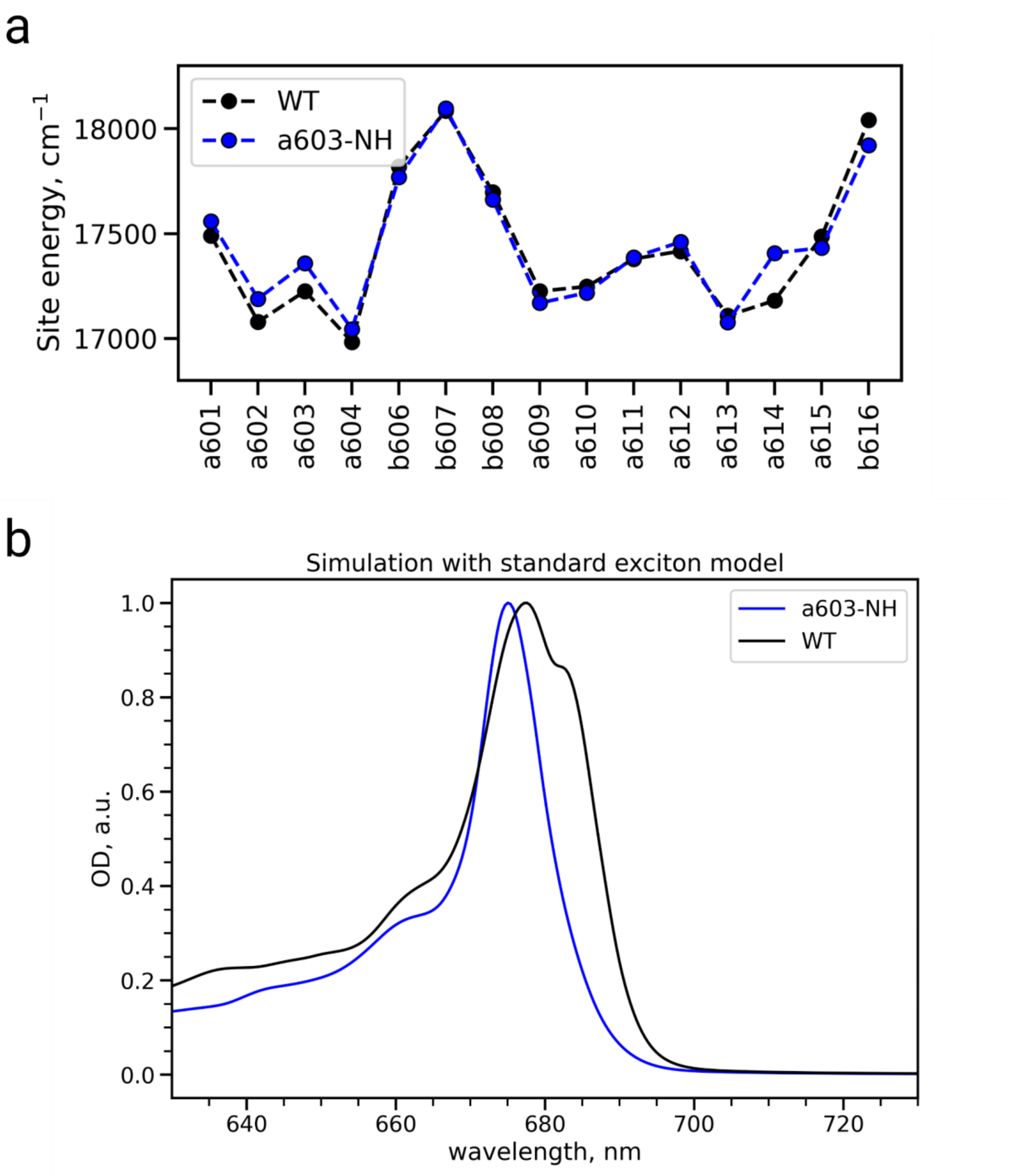

Figure S22: Site energies and simulated absorption spectra. (a) Site energies of Lhca4 Chls for WT and *a*603-NH mutant. (b) Absorption spectra of WT and mutant were simulated using the simple exciton model (local excitations only, without including the CT states). The simulated spectra were rigidly shifted by -1800 cm^{-1} to account for TD-DFT systematic error.

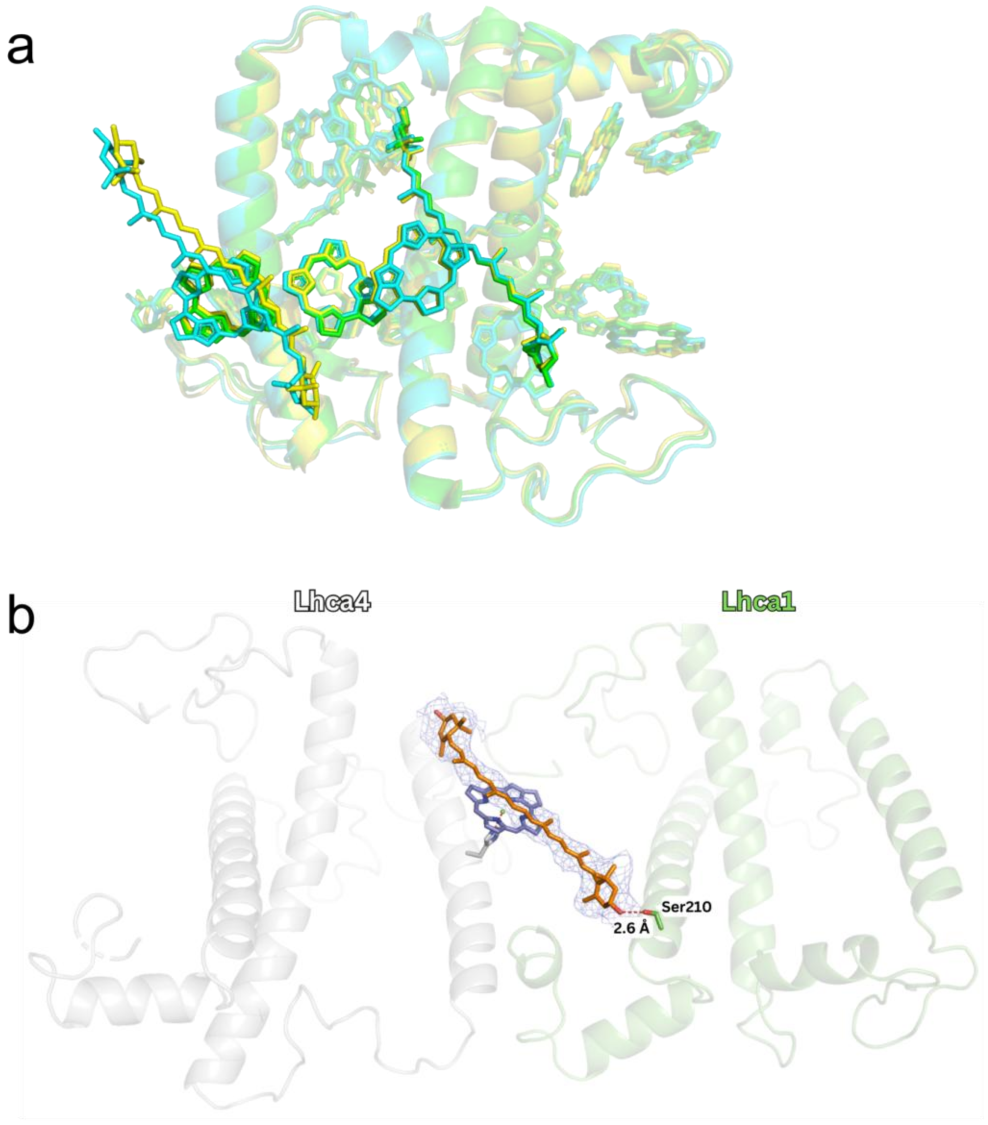

Figure S23: Structural analysis of the Chl *a*615-Lutein cluster. (a) Structural superposition of Lhca4 WT from *A. thaliana* (cyan, PDB 9GBI), *P. sativum* (green, PDB 5L8R), *Z. mays* (yellow, PDB 5ZJI). The "red cluster" formed by Chl *a*603, Chl *a*609, and Chl *a*615 is shown from right to left. The extra lutein is found in the vicinity of Chl *a*615 only in *A. thaliana* and *Z. mays*. (b) Cryo-EM map for Lut over Chl *a*615 in Lhca4. Focus on the hydrogen bond of the Lut hydroxyl with Ser210 from Lhca1.

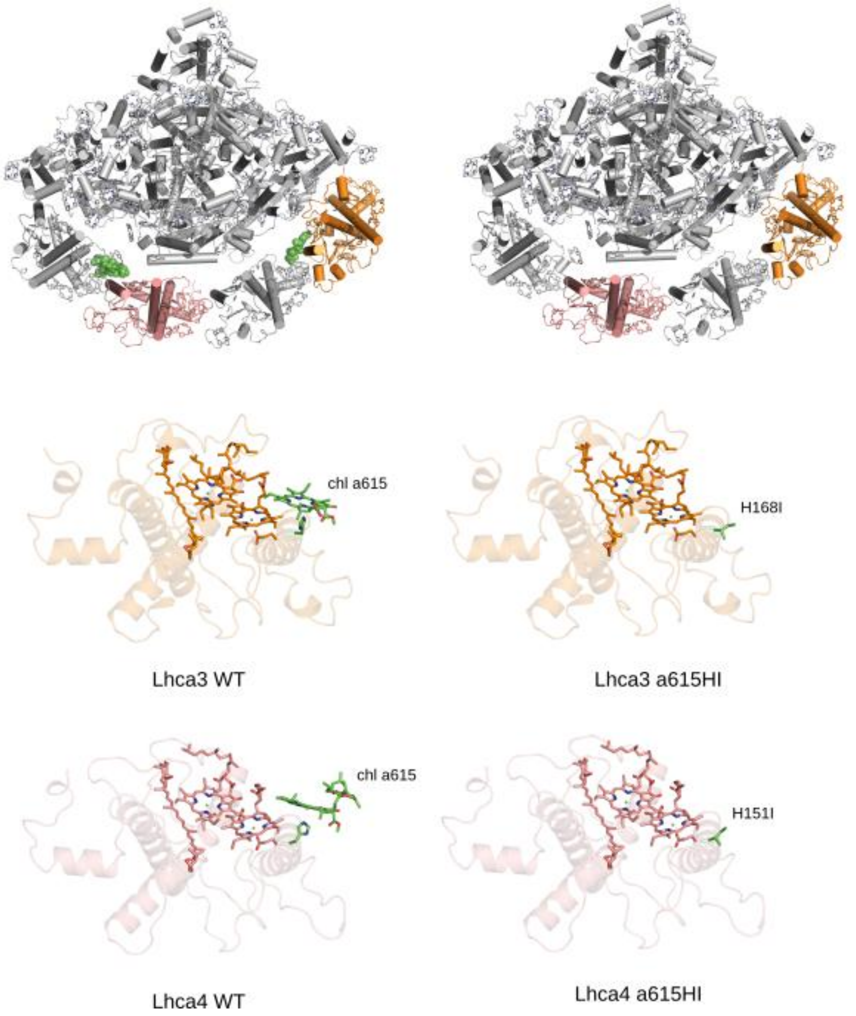

Figure S24: Structural diagram of PSI-LHCI supercomplex, Lhca3 and Lhca4 from WT and the *a*615-HI line. Chls *a*615 and the mutated residues are shown in green. Deletion of Chls and residue substitutions were obtained by *in silico* mutagenesis with PyMol.

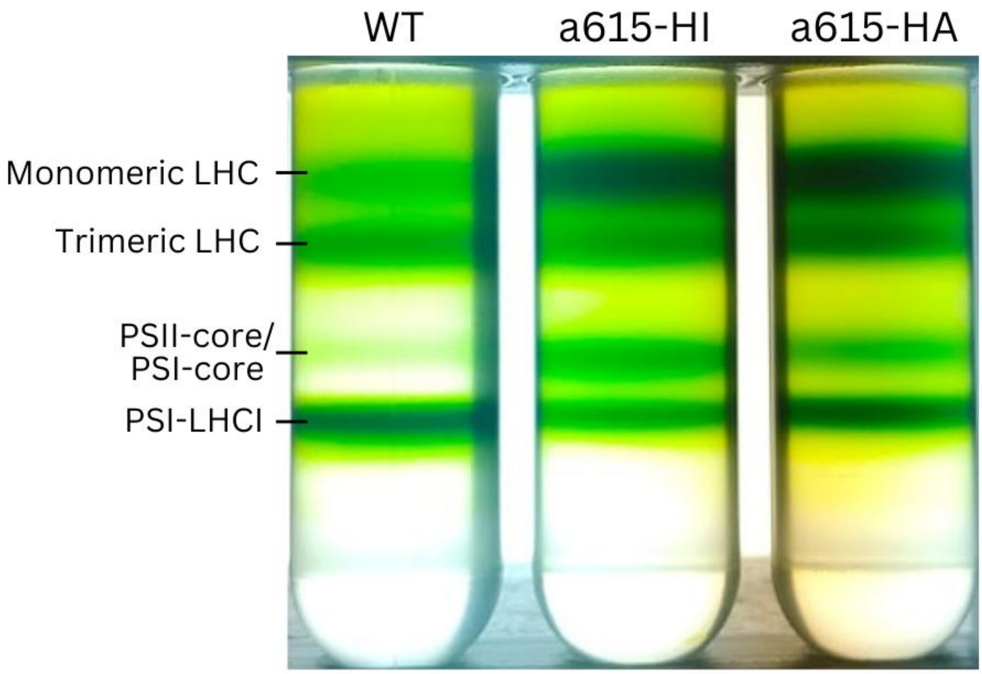

Figure S25: Sucrose gradient fractionation of thylakoid membranes of WT and Chl *a*615 mutant lines solubilized with 1% β-DM.

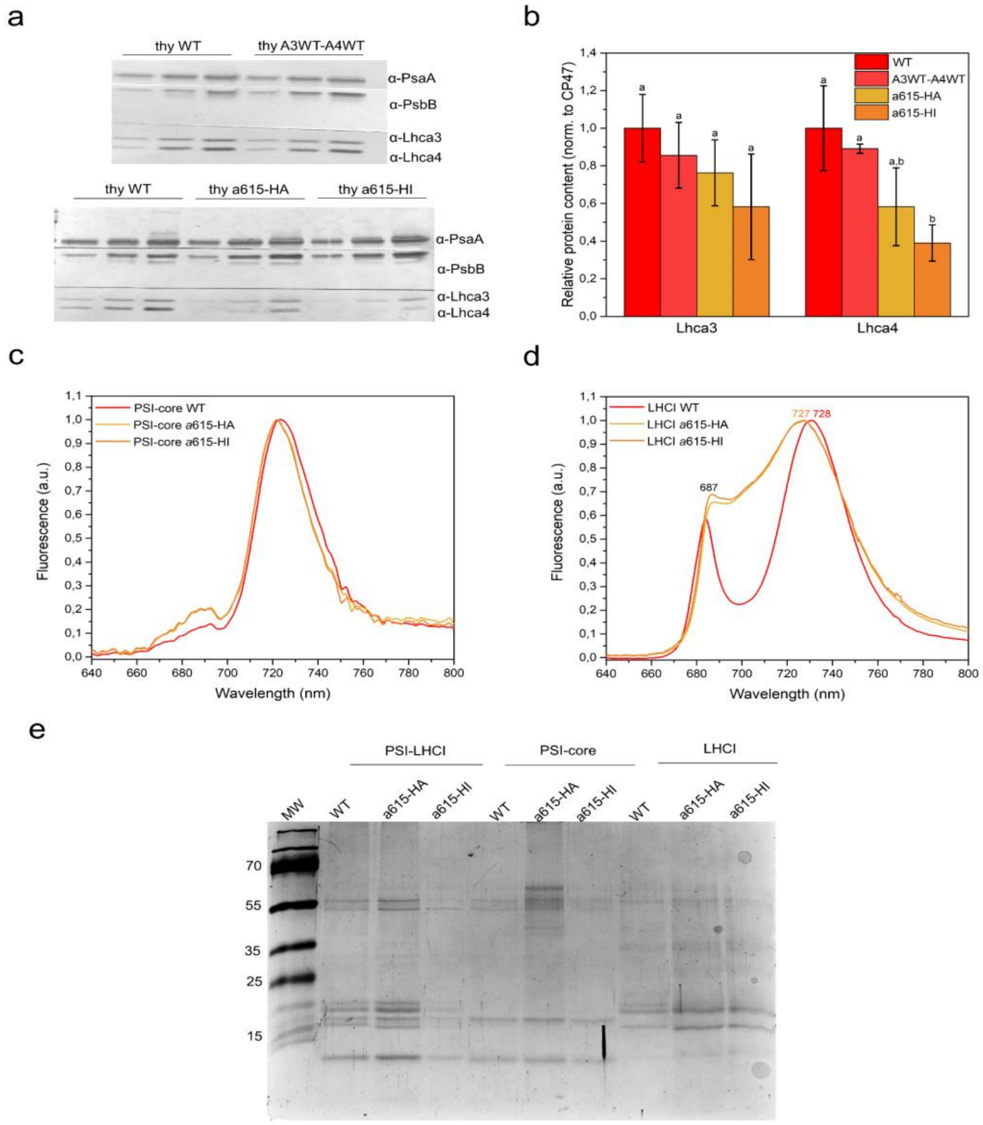

Figure S26: Characterization of Chl *a*615 mutant lines (a) Immunoblotting against PsaA (PSI-core). PsbB (CP47, PSII-core), Lhca3 and Lhca4. (b) Quantification of the relative content of Lhca3 and Lhca4 in *a*615 mutant lines. Values that are significantly different (ANOVA followed by Tukey's post-hoc test at a significance level of $P < 0.05$) are marked with different letters. (c) Fluorescence emission spectra (77K) of PSI-core purified from WT and *a*615-HA and *a*615-HI lines. (d) Fluorescence emission spectra (77K) of LHCI dimers purified from WT and *a*615-HA and *a*615-HI lines. (e) SDS-PAGE analysis of sucrose bands fractions purified from WT and *a*615-HA and *a*615-HI.

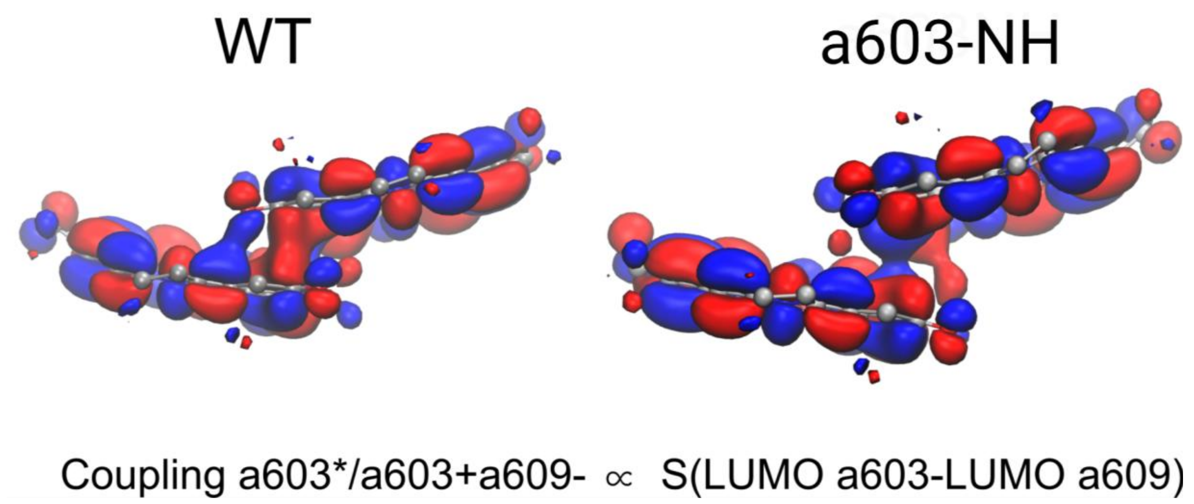

Figure S27: Visual representation of the overlap between LUMO orbitals of Chls *a*603 and *a*609 as computed for the WT and *a*603-NH structures (isovalue +/-0.015).

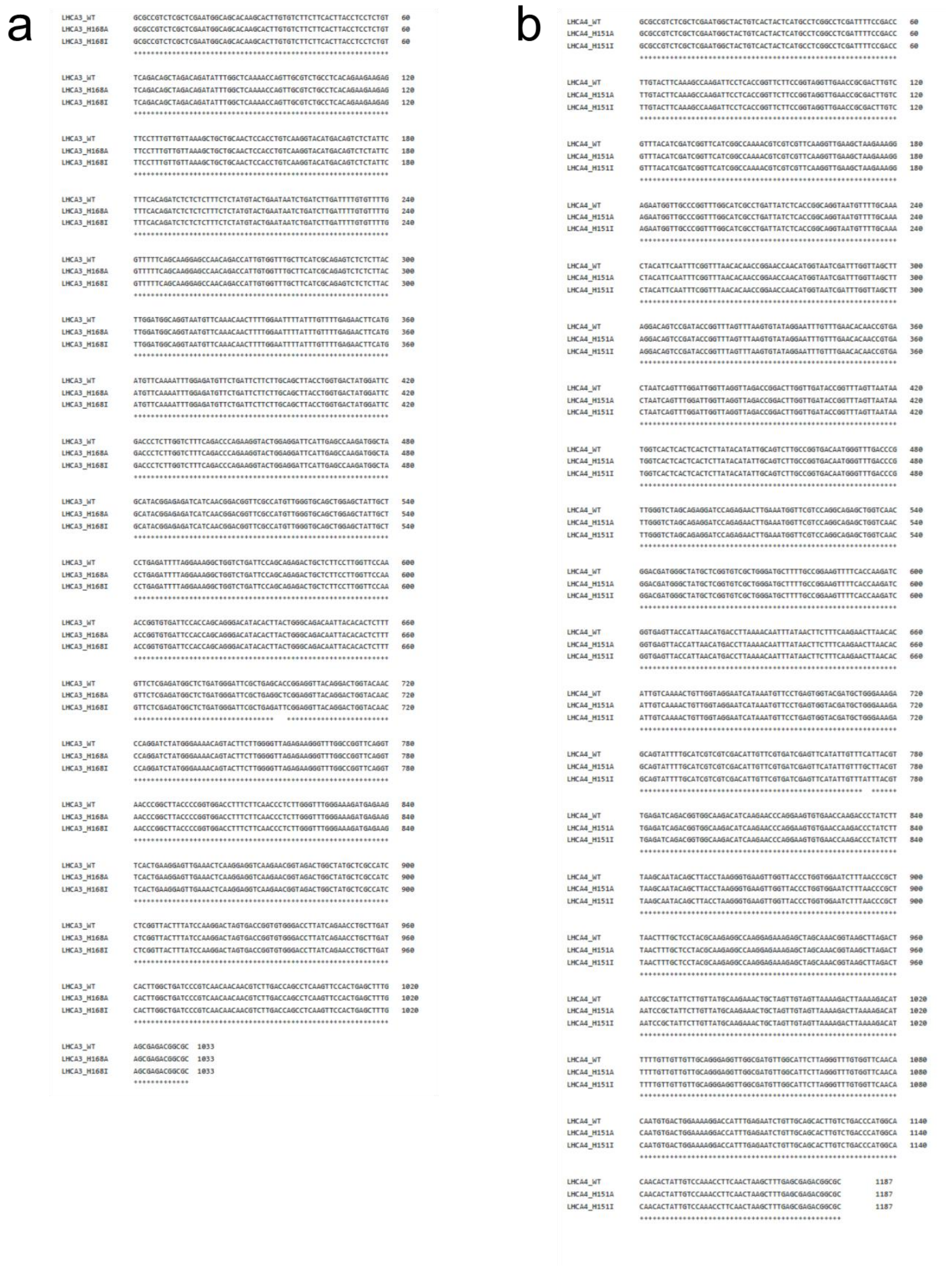

Figure S28: Alignment of the sequences (exons and introns) of the synthetic genes encoding for: (a) Lhca3_WT, Lhca3_a615-H168A and Lhca3_a615-H168I; (b) Lhca4_WT, Lhca4_a615-H151A and Lhca4_a615-H151I.

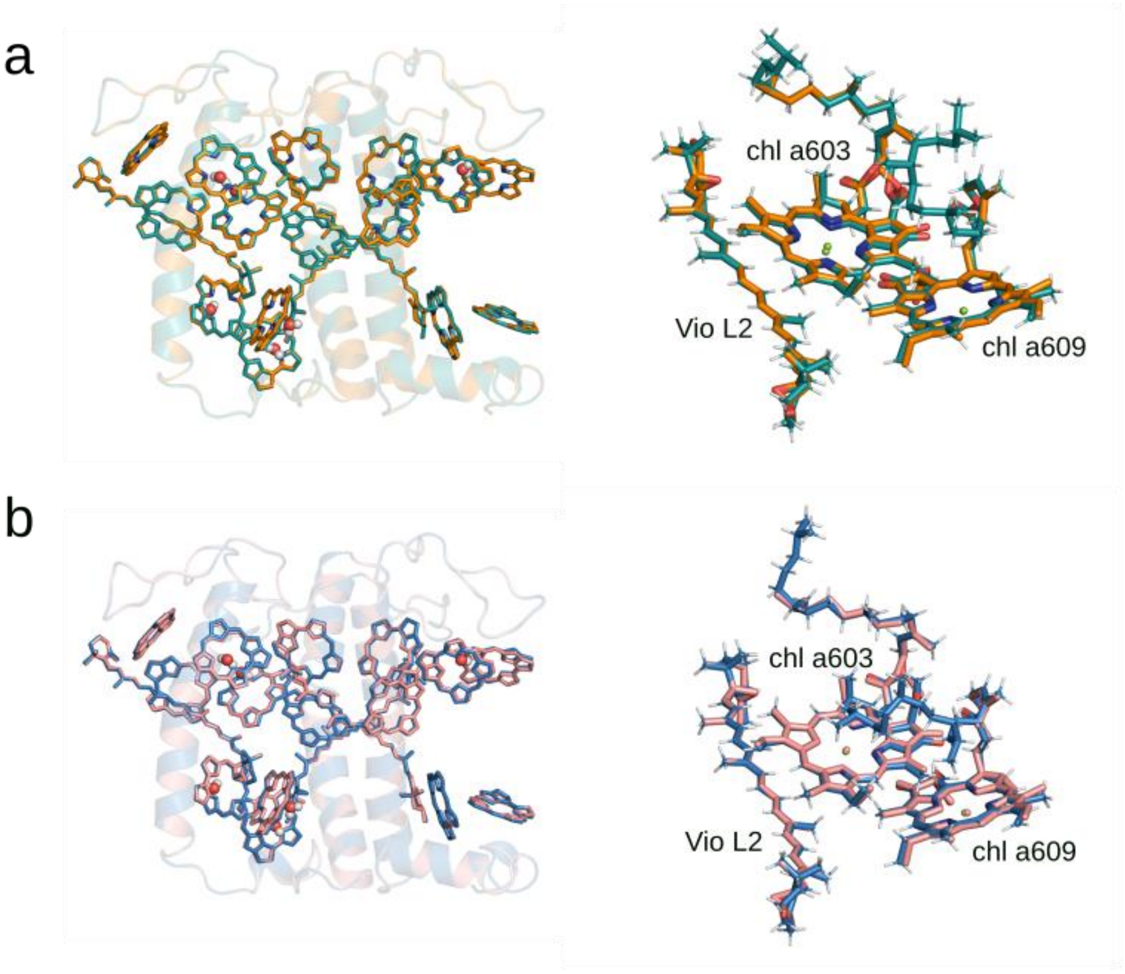

Figure S29: Superposition of Cryo-EM structures of Lhca4 WT (a) or *a*603-NH mutant (b) with the optimized models used for QM/MM calculations. The experimental models are shown in orange and pink, and the optimized structure in aquamarine and blue, respectively. The six water molecules added to the optimized structures are shown as spheres. The conformation of pigments in the Chl *a*603 - Chl *a*609 - Vio L2 cluster is shown in the right panels.

Subunit	Traced residues	Chlorophylls *a*	Chlorophylls *b*	carotenoids	Lipids	Others
PsaA	742	41 CLA		7 BCR	1 LHG, 1 LMT	1 PQN, 1 SF4
PsaB	733	41 CLA		6 BCR	1 LHG, 1 LMG, 1 DGD	1 PQN
PsaC	80	-	-	-	-	2 SF4
PsaD	141	-	-	-	-	-
PsaE	63	-	-	-	-	-
PsaF	152	3 CLA	-	3 BCR	1 LHG	-
PsaG	94	3 CLA	-	1 BCR	1 LHG, 1 LMT	-
PsaH	89	1 CLA	-	-	-	-
PsaI	30	-	-	1 BCR	-	-
PsaJ	39	2 CLA	-	2 BCR	-	-
PsaK	50	2 CLA	-	-	-	-
PsaL	144	3 CLA	-	3 BCR	-	
PsaN	48	-	-	-	-	-
Lhca1	194	12 CLA	2 CHL	1 XAT, 1 LUT, 1 BCR	1 LHG	-
Lhca2	202	9 CLA	5 CHL	1 XAT, 1 LUT, 1 BCR	1 LHG	-
Lhca3	216	13 CLA	1 CHL	1 XAT, 1 LUT, 1 BCR	-	-
Lhca4	195	11 CLA	4 CHL	1 XAT, 2 LUT, 1 BCR	1 LMG	-

Table S1: **AtPSI-*a*603-NH structural model**. CLA, Chl *a*; CHL, Chl *b*; BCR, β-carotene; LUT, lutein; XAT, violaxanthin; DGD, digalactosyl-diacyl glycerol (DGDG); LMG, 1,2-distearoyl-monogalactosyl-digliceride; LHG, 11,2-dipalmitoyl-phosphatidyl-glycerol; LMT, dodecyl-β-D-maltoside; PQN, phylloquinone; SF4, Fe4-S4 cluster.

Primer Name	Sequence	Purpose
**Lhca3_N103H_FW**	CATACGGAGAGATCATCCA TGGACGGTTCGCCATG	Site-directed mutagenesis of Lhca3 N103H
**Lhca3_N103H_RV**	CATGGCGAACCGTCCAT GGATGATCTCTCCGTAT G	Site-directed mutagenesis of Lhca3 N103H
**Lhca4_N99H_FW**	GGCAGAGCTGGTCCATG GACGATGGGCTATG	Site-directed mutagenesis of Lhca4 N99H
**Lhca4_N99H_RV**	CATAGCCCATCGTCCAT GGACCAGCTCTGCC	Site-directed mutagenesis of Lhca4 N99H
**Lhca3_seq_FW**	TCTTCTACCCTAACATAG CCCTTGC	Sequencing of Lhca3 genomic sequence
**Lhca3_seq_RV**	AGAAGCCAATGGTAGCG AAAGATGG	Sequencing of Lhca3 genomic sequence
**Lhca4_seq_FW**	CCAATCCATTCTTCTTCA AGTGCC	Sequencing of Lhca4 genomic sequence
**Lhca4_seq_RV**	TCACAGACAGACATGAA AGTGATGG	Sequencing of Lhca4 genomic sequence

Table S2: List of the primers used to obtain and characterize the *a603-NH* mutant lines.

	AtPSI-WT	AtPSI-603-NH
**Data collection and processing**		
Magnification	120,000X	120,000X
Voltage (kV)	200	200
Total electron dose (e-/Å)	40	40
Nominal defocus range （μm）	0.8 - 2.4	0.8 - 2.4
Pixel size （Å）	0.889	0.889
Symmetry	C1	C1
Initial particle images (no.)	575,271	115,589
Final particle images (no.)	54,155	36,596
Map resolution (Å)	3.13	3.29
FSC threshold	0.143	0.143
**Refinement**		
Initial model used (PDB code)	7DKZ	9GBI
Map sharpening B factor ($Å^2$)	83.7	91.8
Model composition		
Protein residues	3230	3190
Ligands	210	206
B factor ($Å^2$)		
Protein	51.02	70.22
Ligands	49.35	68.56
R.m.s. deviations		
Bond lengths (Å)	0.002	0.003
Bond angles (°)	0.518	0.551
**Validation**		
MolProbity score	1.51	1.48
Clash score	7.19	7.53
Rotamers outliers (%)	0.34	0.27
Ramachandran plot		

Favored (%)	97.4	97.7
Allowed (%)	2.6	2.3
Disallowed (%)	0	0
CC (model vs data)		
Mask	0.88	0.88
Box	0.69	0.72

Table S3: Cryo-EM data collection, refinement, and validation statistics.

	WT	*a*603-NH
*a*603	17320	17207
*a*609	17131	17319
avg Qy	17226	17263
*a*603+*a*609- (Δ CT-Qy)	19486 (+2261)	21318 (+4054)
*a*603-*a*609+ (Δ CT-Qy)	28273 (+11047)	27759 (+10495)
*a*603/*a*609	238	123
*a*603+*a*609-/*a*603	-924	230
*a*603+*a*609-/*a*609	656	-194
*a*603-*a*609+/*a*603	701	-112
*a*603-*a*609+/*a*609	-1074	120

Table S4: **Energies and couplings of the Q_Y and CT states of WT and *a*603-NH**.

Supplemental References

Allen MM, Stanier RY. **1968**. Growth and division of some unicellular blue-green algae. *J Gen Micro.* **51**: 199–202.

Altschul SF, Gish W, Miller W, Myers EW, Lipman DJ. **1990**. Basic local alignment search tool. *Journal of molecular biology* **215**: 403–410.

Ballottari M, Govoni C, Caffarri S, Morosinotto T. **2004**. Stoichiometry of LHCI antenna polypeptides and characterization of gap and linker pigments in higher plants Photosystem I. *European Journal of Biochemistry* **271**: 4659–4665.

Casazza AP, Tarantino D, Soave C. **2001**. Preparation and functional characterization of thylakoids from Arabidopsis thaliana. *Photosynth.Res.* **68**: 175–180.

Case DA, Belfon K, Ben-Shalom IY, Brozell SR, Cerutti DS, Cheatham TE, Cruzeiro VWD, Darden T, Duke RE, Giambasu G, *et al.* **2020**. AMBER2020, University of California, San Fransisco. *J. Amer. Chem. Soc* **142**: 3823–3835.

Chung LW, Sameera WMC, Ramozzi R, Page AJ, Hatanaka M, Petrova GP, Harris T V, Li X, Ke Z, Liu F, *et al.* **2015**. The ONIOM method and its applications. *Chemical reviews* **115**: 5678–5796.

Frisch MJ, Trucks GW, Schlegel HB, Scuseria GE, Robb MA, Cheeseman JR, Scalmani G, Barone V, Petersson GA, Nakatsuji H, *et al.* **2016**. Gaussian 16, revision a. 03, gaussian, inc., wallingford ct. *Gaussian16 (Revision A. 03).*

Gelzinis A, Abramavicius D, Valkunas L. **2015**. Absorption lineshapes of molecular aggregates revisited. *The Journal of Chemical Physics* **142**.

Kumar S, Suleski M, Craig JM, Kasprowicz AE, Sanderford M, Li M, Stecher G, Hedges SB. **2022**. TimeTree 5: an expanded resource for species divergence times. *Molecular biology and evolution* **39**: msac174.

Laemmli UK. **1970**. Cleavage of structural proteins during the assembly of the head of bacteriophage T4. *Nature*.

Madeira F, Pearce M, Tivey ARN, Basutkar P, Lee J, Edbali O, Madhusoodanan N, Kolesnikov A, Lopez R. **2022**. Search and sequence analysis tools services from EMBL-EBI in 2022. *Nucleic acids research* **50**: W276--W279.

Madjet ME, Abdurahman A, Renger T. **2006**. Intermolecular Coulomb couplings from ab initio electrostatic potentials: application to optical transitions of strongly coupled pigments in photosynthetic antennae and reaction centers. *The Journal of Physical Chemistry B* **110**: 17268–17281.

Nottoli M, Jurinovich S, Cupellini L, Gardiner AT, Cogdell R, Mennucci B. **2018**. The role of charge-transfer states in the spectral tuning of antenna complexes of purple bacteria. *Photosynthesis research* **137**: 215–226.

Novoderezhkin VI, Palacios MA, Van Amerongen H, Van Grondelle R. **2004**. Energy-transfer dynamics in the LHCII complex of higher plants: modified redfield approach. *The Journal of Physical Chemistry B* **108**: 10363–10375.

Pinnola A, Alboresi A, Nosek L, Semchonok D, Rameez A, Trotta A, Barozzi F, Kou\vril R, Dall'Osto L, Aro E-M, *et al.* 2018. A LHCB9-dependent photosystem I megacomplex induced under low light in Physcomitrella patens. *Nature Plants* **4**: 910–919.

Prandi IG, Viani L, Andreussi O, Mennucci B. **2016**. Combining classical molecular dynamics and quantum mechanical methods for the description of electronic excitations: the case of carotenoids. *Journal of computational chemistry* **37**: 981–991.

Saraceno P, Sláma V, Cupellini L. **2023**. First-principles simulation of excitation energy transfer and transient absorption spectroscopy in the CP29 light-harvesting complex. *The Journal of Chemical Physics* **159**.

Sarrion-Perdigones A, Vazquez-Vilar M, Palací J, Castelijns B, Forment J, Ziarsolo P, Blanca J, Granell A, Orzaez D. **2013**. Goldenbraid 2.0: A comprehensive DNA assembly framework for plant synthetic biology. *Plant Physiology* **162**: 1618–1631.

Sláma V, Cupellini L, Mascoli V, Liguori N, Croce R, Mennucci B. **2023**. Origin of Low-Lying Red States in the Lhca4 Light-Harvesting Complex of Photosystem I. *Journal of Physical Chemistry Letters* **14**: 8345–8352.

Sláma V, Cupellini L, Mennucci B. **2020**. Exciton properties and optical spectra of light harvesting complex II from a fully atomistic description. *Physical Chemistry Chemical Physics* **22**: 16783–16795.

Sueoka N. **1960**. MITOTIC REPLICATION OF DEOXYRIBONUCLEIC ACID IN CHLAMYDOMONAS REINHARDI. *Proceedings of the National Academy of Sciences*.

Vazquez-Vilar M, Quijano-Rubio A, Fernandez-del-Carmen A, Sarrion-Perdigones A, Ochoa-Fernandez R, Ziarsolo P, Blanca J, Granell A, Orzaez D. **2017**. GB3. 0: a platform for plant bio-design that connects functional DNA elements with associated biological data. *Nucleic acids research* **45**: 2196–2209.

Wang J, Yu L-J, Wang W, Yan Q, Kuang T, Qin X, Shen J-R. **2021**. Structure of plant photosystem I- light harvesting complex I supercomplex at 2.4 Å resolution. *Journal of Integrative Plant Biology* **63**: 1367–1381.
